# Exercise Ameliorates Motor Deficits and Improves Dopaminergic Functions in the Rat Hemi-Parkinson’s Model

**DOI:** 10.1038/s41598-018-22462-y

**Published:** 2018-03-05

**Authors:** Yuan-Hao Chen, Tung-Tai Kuo, Jen-Hsin Kao, Eagle Yi-Kung Huang, Tsung-Hsun Hsieh, Yu-Ching Chou, Barry J. Hoffer

**Affiliations:** 1 Department of Neurological Surgery, Tri-Service General Hospital, National Defense Medical Center, Taipei, Taiwan, R.O.C.; 2 Graduate Institute of Computer and Communication Engineering, National Taipei University of Technology, Taipei, Taiwan, R.O.C.; 3 Department of Pharmacology, National Defense Medical Center, Taipei, Taiwan, R.O.C.; 4 Department of Physical Therapy and Graduate Institute of Rehabilitation Science, Chang Gung University, Taoyuan, Taiwan; 5 School of Public Health, National Defense Medical Center, Taipei, Taiwan, R.O.C.; 6 Graduate Program on Neuroregeneration, Taipei Medical University, Taipei, Taiwan; 7 Department of Neurosurgery, Case Western Reserve University School of Medicine, Cleveland, Ohio, USA

## Abstract

To determine the influences of exercise on motor deficits and dopaminergic transmission in a hemiparkinson animal model, we measured the effects of exercise on the ambulatory system by estimating spatio-temporal parameters during walking, striatal dopamine (DA) release and reuptake and synaptic plasticity in the corticostriatal pathway after unilateral 6-OHDA lesions. 6-OHDA lesioned hemiparkinsonian rats were exercised on a fixed speed treadmill for 30 minutes per day. Controls received the same lesion but no exercise. Animals were subsequently analyzed for behavior including gait analysis, rotarod performance and apomorphine induced rotation. Subsequently, *in vitro* striatal dopamine release was analyzed by using FSCV and activity-dependent plasticity in the corticostriatal pathway was measured in each group. Our data indicated that exercise could improve motor walking speed and increase the apomorphine-induced rotation threshold. Exercise also ameliorated spatiotemporal impairments in gait in PD animals. Exercise increased the parameters of synaptic plasticity formation in the corticostriatal pathway of PD animals as well as the dynamics of dopamine transmission in PD animals. Fixed speed treadmill training 30 minutes per day could ameliorate spatial-temporal gait impairment, improve walking speed, dopamine transmission as well as corticostriatal synaptic plasticity in the unilateral 6-OHDA lesioned rat model.

## Introduction

Parkinson’s disease is second most common neuron degenerative disease, and is characterized by symptoms related to progressive dopamine neuron loss within the substantia nigra pars compacta^1^. In addition to dopaminergic neuron loss, other neurotransmitter systems have been indicated to be involved in the disease; thus PD is now been thought to be multisystem disorder^2,3^.

Gait disturbances have been shown in Parkinson’s disease (PD)^4^. In addition to the typical motor deficits in Parkinson’s disease including bradykinesia, rigidity, resting tremor and postural instability, increasing data has indicated that incoordination and temporal asymmetry results in disturbances of internal gait rhythm and walking speed in PD subjects^5^. The cadence and double stand time also increases^4^. Moreover, the deficits in spatial indices of gait seen in PD patients and animals includes short steps^6^, decreased stride length and freezing^7^. These abnormalities in gait increased as the disease progressed^8^.

Exercise is currently an important treatment for PD^9,10^. Not only the motor symptoms but also a myriad of nonmotor symptoms may present in PD patients. Although both motor and nonmotor symptoms may affect PD patients’ ability to participate in exercise and/or impact the outcomes of exercise, PD patients still have the ability to participate in many forms of exercise and generally respond to exercise interventions similar to subjects of matching age without PD. Therefore, exercise has currently become an area of increased research to investigate the mechanisms through which the exercise may affect the progression of the disease.

Increasing evidence also indicates that exercise improves the PD motor symptoms and reduces dopaminergic neuron loss in PD animal models^11,12^. This may involve several mechanisms, including upregulation of neurogenesis^13^, angiogenesis^14^ and enhanced neuronal plasticity^15,16^. In addition, neurotrophic factors that play a crucial role in changes in brain plasticity and neurogenesis are induced by exercise^17^.

Hsieh *et al*., have shown that hemiparkinsonian rats exhibit changes in gait patterns with significantly decreased walking speed, decreased step/stride length and increased base of support and foot angle^8^. Moreover, neuroplasticity has been indicated impaired in PD animals and patients^18^.

In this paper, we determined the relationship between gait deficits and corticostraital plasticity impairment related to dopamine degeneration and with respect to exercise. We assessed spatio-temporal gait analysis, dopamine release via fast cycle voltammetry (FSCV) and corticostriatal synaptic plasticity in the 6-OHDA lesioned hemiparkinson rat and how these parameters were influenced by exercise.

## Results

### Exercise improved motoric walking speed and increased the apomorphine –induced rotation threshold in PD animals

**Figure 1:**
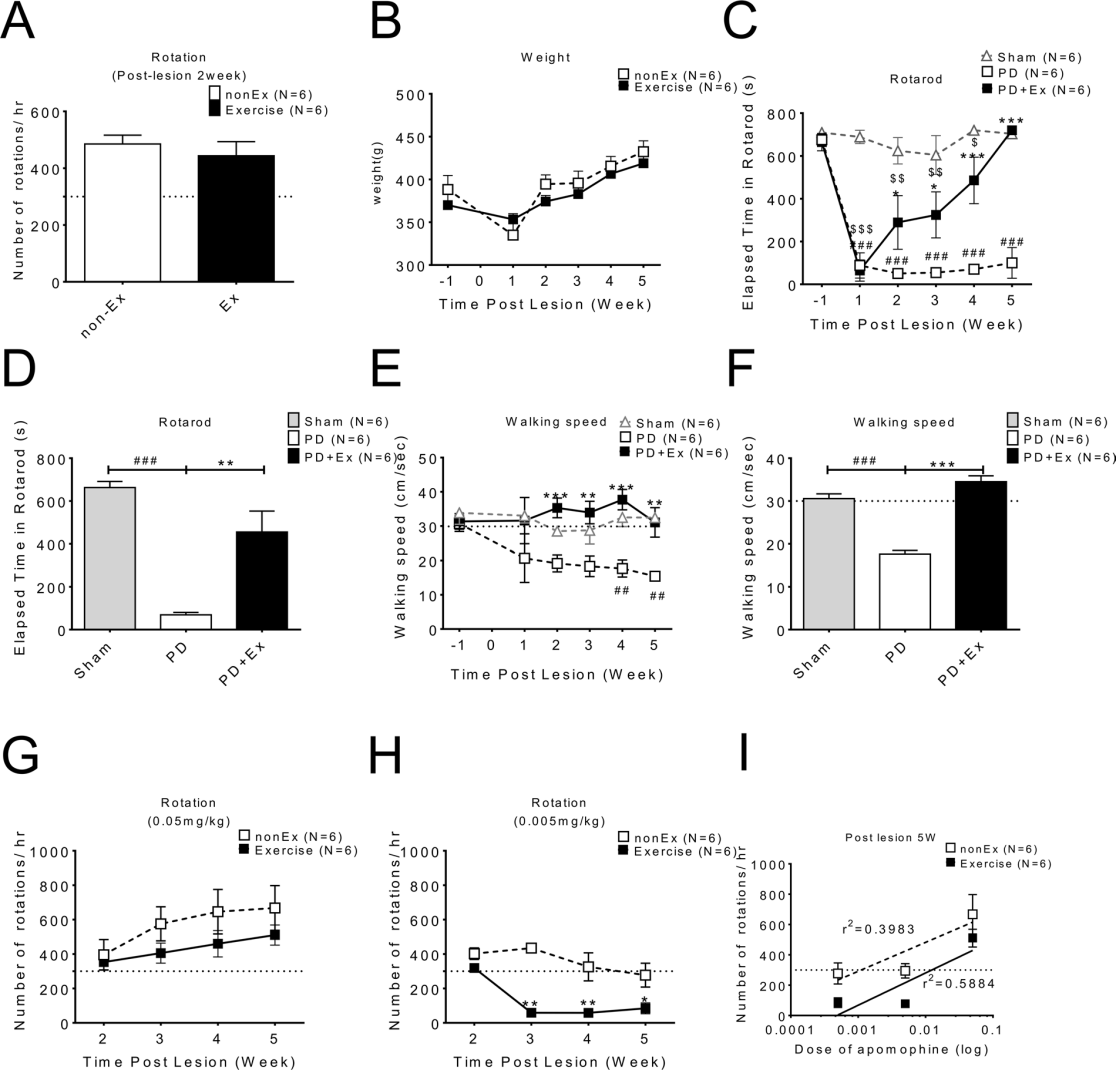
Exercise could improve walking speed, rotarod coordination and reduce supersensitivity of apomorphine –induced rotation in PD animals. Motor behavior tests were performed for 4 weeks after 6-OHDA lesions and data were obtained from a comparison between PD and PD with exercise animals. (**A**) Apomorphine-induced rotation was used to confirm that the 6-OHDA lesion induced hemi-parkinsonism; there was no significant difference between PD and PD with exercise with apomorphine initially. (**B**) There were no differences in body weight between groups. (**C**) The rotarod test revealed that the short retention time in PD animals could be lengthened by exercise. (**D**) The data from the rotarod of each group from post-lesion 2nd to 5th weeks were averaged and ploted. (**E**) The walking speed improved gradually during exercise training and significant improvement could be found after four-weeks of exercise. (**F**) The data on walking speed of each group from post-lesion 2nd to 5th weeks were averaged and ploted. (**G–H**) the apomorphine-induced rotation data showing reduced supersensitivity in the PD exercise group.

Apomorphine rotation initially used to confirm a hemiparkinson’s animal model after the 6-OHDA lesion. Treadmill running were performed 30 minutes per day in the PD with exercise group beginning the 1st week after the 6-OHDA lesion. Motor tests, including walking speed and rotarod were performed subsequently weekly for 4 weeks from post-lesion 2^nd^ to 5^th^ weeks, and data were obtained from PD and PD with exercise animals (Fig. 1). There were no significant difference in initial apomorphine-induced rotation (Fig. 1A) after 6-OHDA lesions and in body weight (Fig. 1B) between PD and PD with exercise groups. Elapsed retention time on the rotarod is a measure of motor coordination, and with repeated trials, also a measure of motor learning. PD animals had a very short retention time on the rotarod which did not improve with subsequent trials. In contrast, 2 weeks after the lesion, the PD exercise group had a significantly longer retention time, with further increases post-lesion 3rd to 5th weeks (Fig. 1C). The averaged rotarod data from each group from post-lesion 2nd to 5th weeks revealed significant improvement in the PD with exercise group (Fig. 1D). Walking speed were also measured and indicated that exercise significantly improved walking speed in PD animals (Fig. 1E,F). Apomorphine-induced rotation could be generated by dosage of 0.05 mg/kg in both PD and PD with exercise groups (Fig. 1G). But with dosage of 0.005 mg/kg, apomorphine induced rotation only were seen in PD rather than in the PD with exercise group (Fig. 1H) which suggests reduced supersensitivty.

### Exercise ameliorates spatiotemporal impairments in gait in PD animals

**Figure 2:**
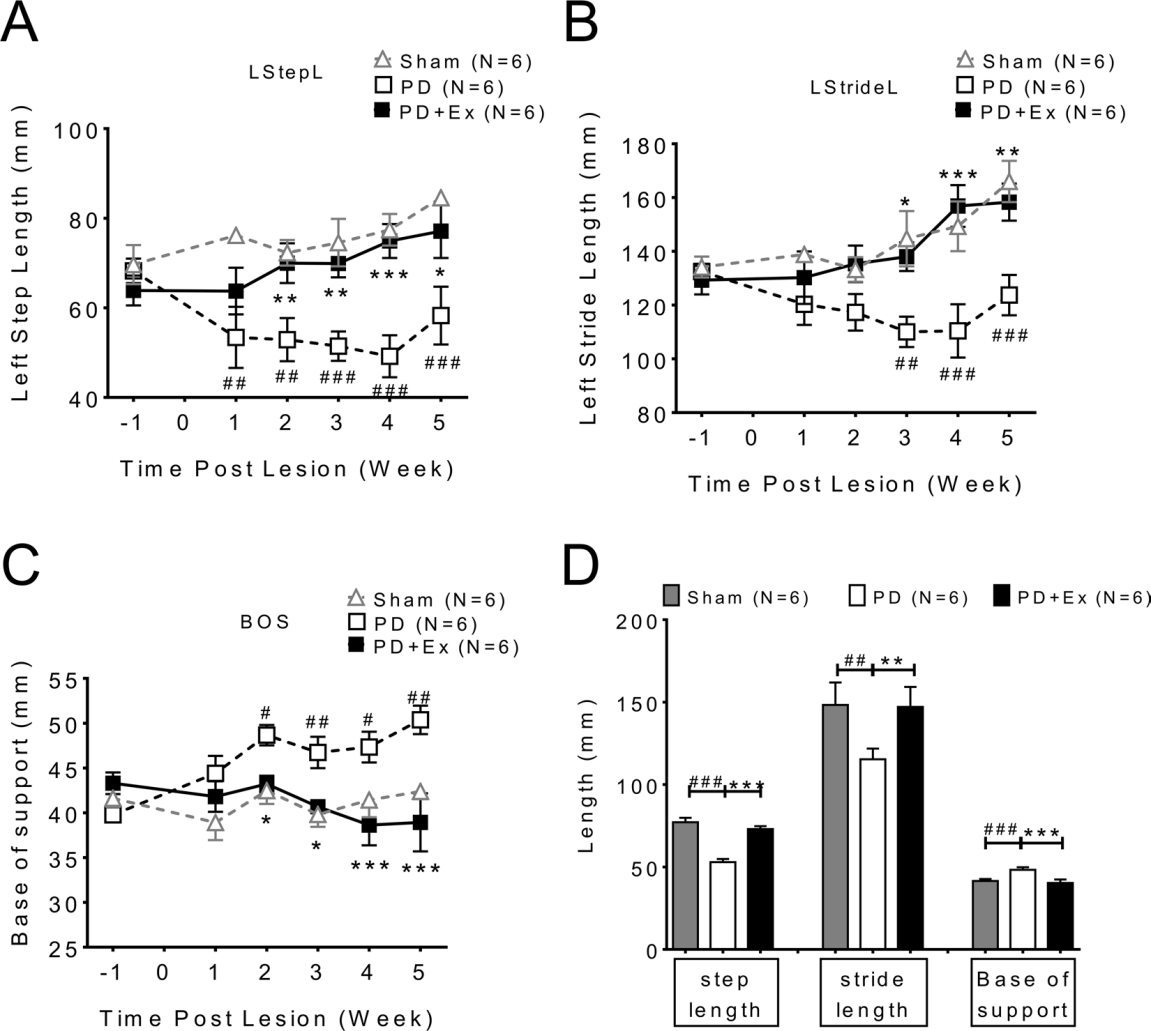
The spatial support parameters between limbs of PD animal improved after exercise. (**A**) Base of support, (**B**) instep length (StepL) and (**C**) stride length (StideL) on the lesioned side limb (left) revealed significant improvements during 4 weeks of exercise after the 6-OHDA lesion. (**D**) The bar chart shows average post-lesion 2nd to 5th week’s data from each group.

As previous data reported^8^, our data also indicated that spatial support parameters between limbs including base of support, step length and stride length in PD animals were longer than in the sham group (Fig. 2A~C, PD: open square vs. sham: open triangle). However, after 4 weeks of exercise, these parameters improved gradually. (Fig. 2A~C, PD: open square vs. PD + exercise: solid square). The averaged post-lesion 2^nd^ to 5^th^ week’s data from each group revealed improvement of hind limb support in the exercise group (Fig. 2D).

**Figure 3:**
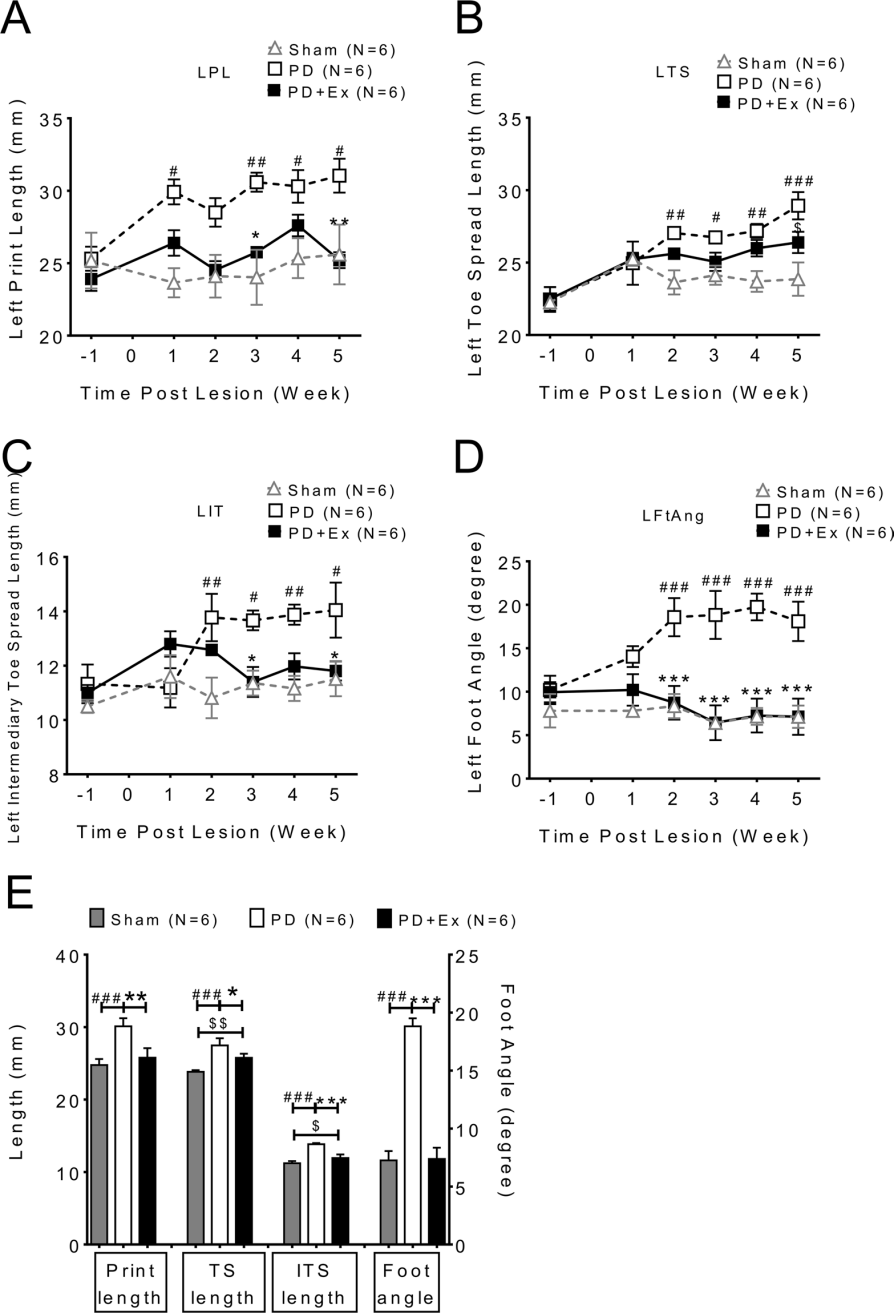
The support parameters of paw and hind limb were improved in PD with exercise animal. (**A**) Print length, (**C**) intermediate toe spread and (**D**) foot angle data revealed impairments were ameliorated after exercise in PD animals. However, (**B**) toe spread length was similar between groups. (**E**) The data from support parameters of each group from post-lesion 2nd to 5th weeks were averaged and plotted. (PL: print length, TS: Toe spread, IT: Intermediate toe spread, Ft Ang: Foot angle).

The support parameters of paws on affected (left) hind limb, including print length (PL), toe spread length (TS), intermediate toe spread (IT) and foot angle (FA) were also affected by 6-OHDA, one week after the 6-OHDA lesion (Fig. 3A~D lesion side (left) hind limb, PD: open square vs. sham: open triangle, two way ANOVA followed by Bonferroni post-hoc test with significance levels <0.05#, <0.01## and <0.005###). The PD exercise animals had gradually improved support parameters especially in print length, intermediate toe spread and foot angle (Fig. 3A~D, PD: open square vs. PD with exercise: solid square, two way ANOVA followed by Bonferroni post-hoc test with significance levels <0.05*, <0.01** and <0.005***). The toe spread lengths were not different between PD and PD with exercise initially but improved at 4 weeks of exercise (Fig. 3B). The averaged data of support parameters for each group from post-lesion 2^nd^ to 5^th^ weeks showed the deficits in foot support in PD animals were gradually improved after exercise.(Fig. 3E)

**Figure 4:**
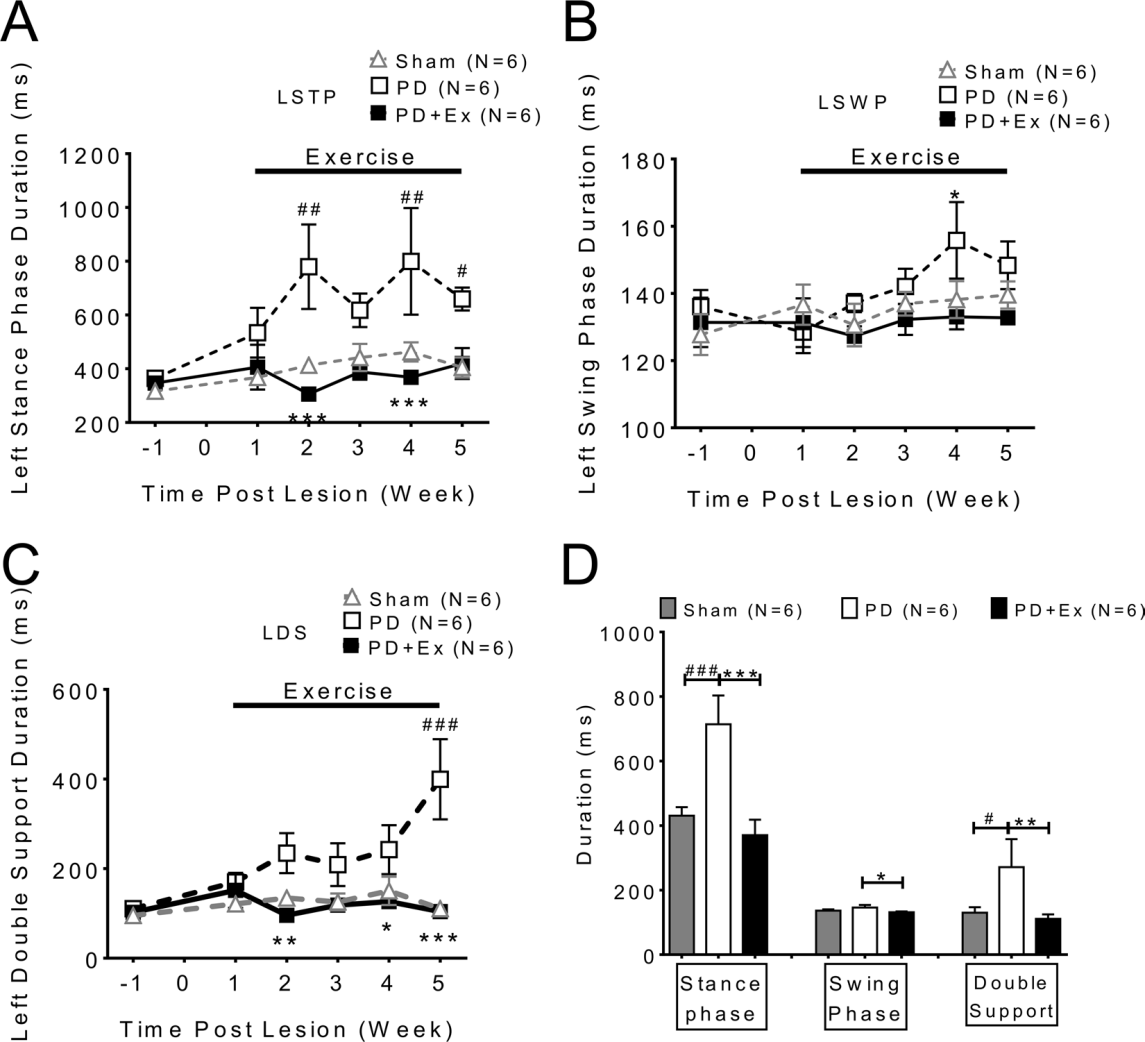
The temporal parameters of gait in PD animals improved after exercise. The temporal indices which include: (**A**) stance time (STP), (**B**) wing phase (SWP) and (**C**) double support (DS) were improved in PD in exercise animals compared with PD only animals. (**D**) The data of each group collected from 2nd to 5th week were averaged; significant improvement of temporal indices was noted in exercise animals (Black column vs white column, one-way ANOVA with a Bonferroni post hoc test).

Temporal parameters of gait including stance time (STP) (Fig. 4A), wing phase (SWP) (Fig. 4B) and double support (DS) (Fig. 4C) were impaired in PD animals as previously described^8^. This temporal gaiting impairment improved significantly after exercise, which is shown in the averaged data from each group from 2^nd^ to 5^th^ weeks (Fig. 4D, PD: white column vs. PD with exercise: black column, two way ANOVA followed by Bonferroni post-hoc test with significance levels indicated in the Figure legend).

**Figure 5:**
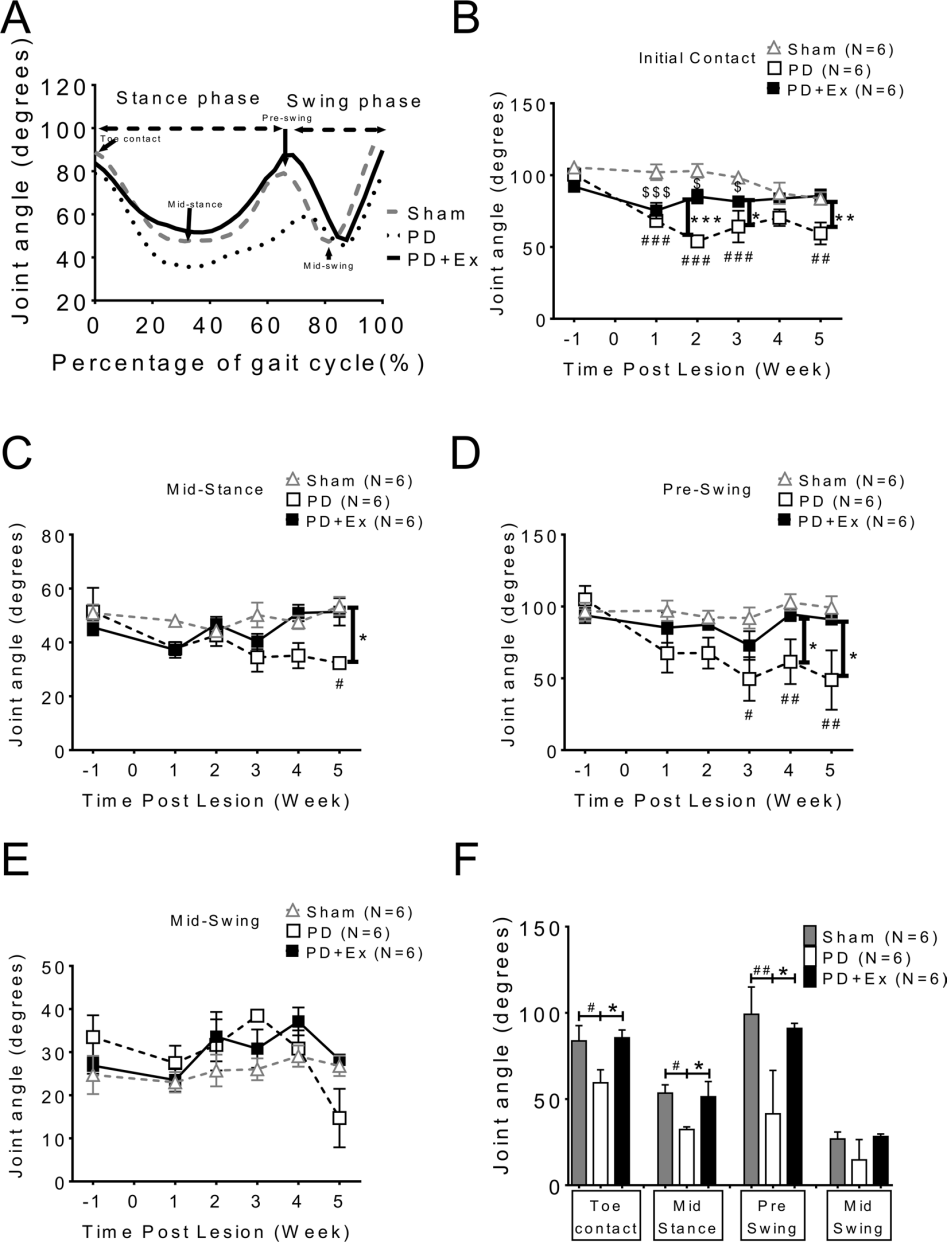
Kinetic parameters examined at four specific stages during locomotion: analysis of dynamic changes in ankle angle during the gait cycle. (**A**) Angular trajectories of the ankle joint of a sham rat (gray dotted line) a PD rat (black dotted line) and PD with exercise rat (solid line) in a full gait cycle were measured. The stance and swing phases were normalized to percentage of a full gait cycle of 100% (X-axis). The arrows indicate the beginning of the stance phase (toe contact), mid-stance, pre-swing, and mid-swing in the gait cycle. Not only in stance phase but also in initial swing phase (pre-swing stage), the ankle angle of PD was smaller than that of sham animals. (**B**) Measurements of ankle joint angles at four specific time points in normal and PD rats. Data are averaged by four weeks’ (post-lesion 2nd to 5th week) and presented as mean ± SEM. *which indicated a significant difference (p < 0.05) between the PD and PD with exercise animals in toe contact, mid-stance and pre-swing phases (unpaired Student t tests).

In addition to temporo-spatial indices of gait, the kinetic ankle angle parameters during the gait cycle at four specific stages were also measured (Fig. 5). Angular trajectories of the ankle joint in a full gait cycle were determined and the stance and swing phases were normalized as a percentage of a full gait cycle of 100% (X-axis). The mid-stance phase and initial swing phase (pre-swing stage) angles in PD were smaller than in sham animals (Fig. 5A representative trace of one animal group: sham: gray dotted line; PD: black dotted line; and PD with exercise: solid line). The averaged data at four weeks (post-lesion 2^nd^ to 5^th^ week) showed significant differences between the PD and PD with exercise animals in toe contact, mid-stance, and pre-swing phases (Fig. 5B, two way ANOVA followed by Bonferroni post-hoc test with significance levels, *p < 0.05 and **p < 0.01). The data on motor performance is summarized in Table 1.

**Table 1:** The summary of the effect on gait in each parameter analysis of the PD animal in our exercise training.

Behavior test and gait parameter	Outcome	Data description
Rotar rod	V	Post-Ex 4w PD & PD + Ex: ***
Rotation		Post-6-OHDA lesion 2w PD& PD + Ex > 300 turns/Hr
Kinetic Indices		
Walking Speed	V	Post-Ex 4w PD& PD + Ex: *
Joint ankle	V	Post-Ex 4w PD& PD + Ex: *
Spatial Indices		
Step length	V	Post-Ex 4w PD& PD + Ex: *
Base of support	V	Post-Ex 4w PD& PD + Ex: **
Stride length	V	Post-Ex 4w PD& PD + Ex: **
Foot angle	V	Post-Ex 4w PD& PD + Ex: **
Print length	V	Post-Ex 4w PD& PD + Ex: **
The intermediary toe spread	X	
Toe spread	V	Post-Ex 4w PD& PD + Ex: *
Temporal Indices		
Stance time	X	
Swing time	X	
Double support	V	Post-Ex 4w PD& PD + Ex: ***

Outcome: V: improved, X: non-improvement.

Interestingly, there was significant improvement in many gait parameters or on the right (healthy) side as shown in Suppl Figs 2–1, 2, 3. This correlates with a previous clinical study in PD subjects showing bilateral improvement after a unilateral striatal graft of fetal DA neurons.

### The effect of exercise on corticostriatal pathway plasticity in PD

**Figure 6:**
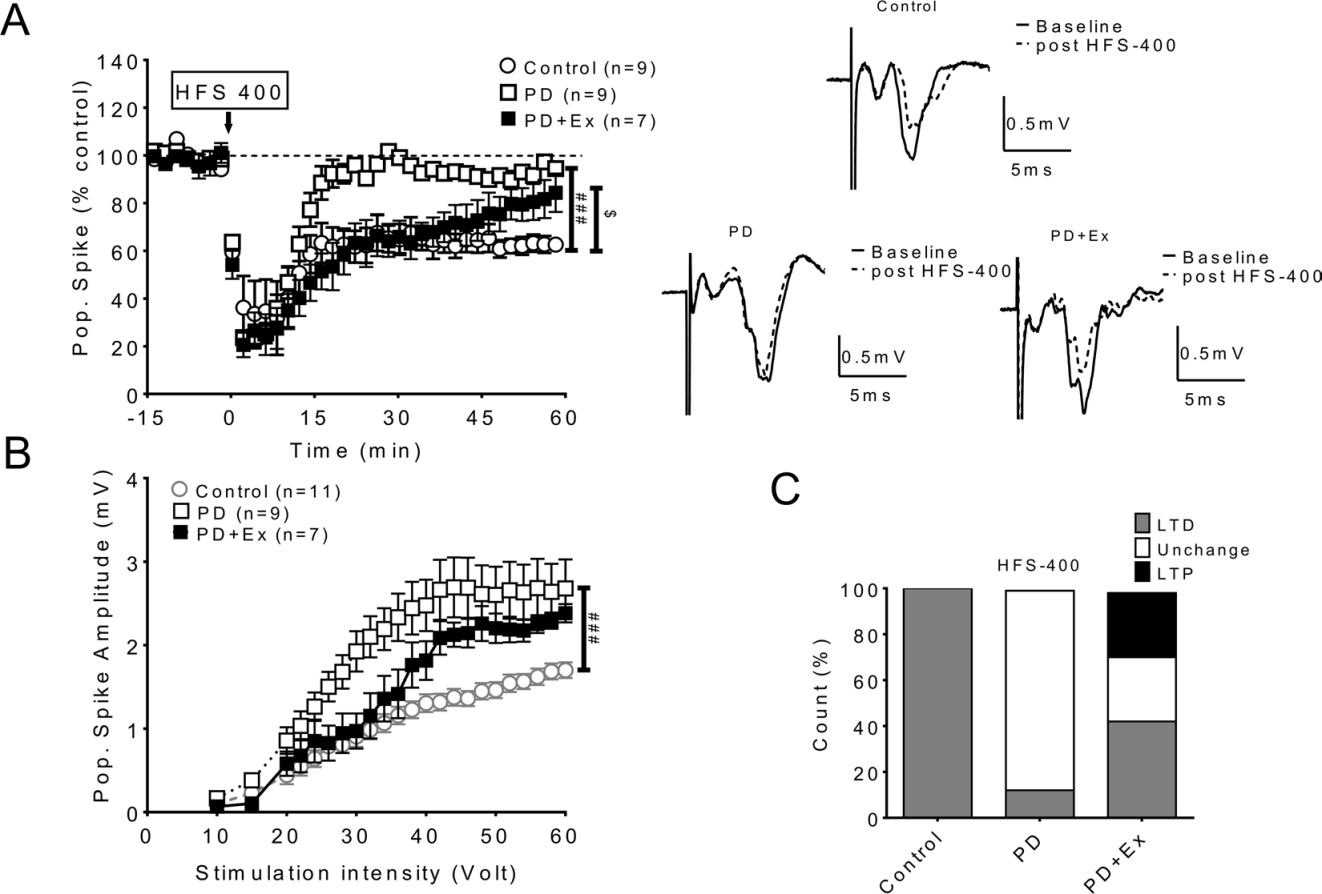
Changes in synaptic plasticity of the corticostriatal pathway. (**A**) LTD was induced in the corticostriatal pathway in control animals (Open circle), while LTD was impaired in 6-OHDA lesioned animals (Open square). Partially recovered LTD could be found in the PD with exercise group, but this plasticity could not be maintained. (**B**) The input/output curve of the corticostriatal pathway, with significant enhancement of glutamatergic population spike in PD but a return to normal in the PD with exercise group under low stimulation intensity and increasing amplitude evoked by higher stimulation intensities. (**C**) The incidence (number) of synaptic plasticity formation in the corticostriatal pathway increased in exercise group compared with PD animals.

The cortico-striatal pathway, which has been suggested to be impaired in PD animals^19–21^, plays the crucial role in skill formation, local motor control and habit formation. We analyzed synaptic plasticity in the corticostriatal pathway in each group. We found that LTD was normally induced in the corticostriatal pathway in control animals (Fig. 6A solid circles), whereas LTD was impaired in 6-OHDA lesioned animals (Fig. 6A open circles). However, after 4weeks of exercise, partial recovery in plasticity could be found initially; however, this plasticity was not maintained (Fig. 6B). The changes in cortico-striatal plasticity in the PD with exercise animals compared with those in other groups are plotted in Fig. 6C.

### The dynamic effect of exercise in dopamine transmission in PD animals

**Figure 7:**
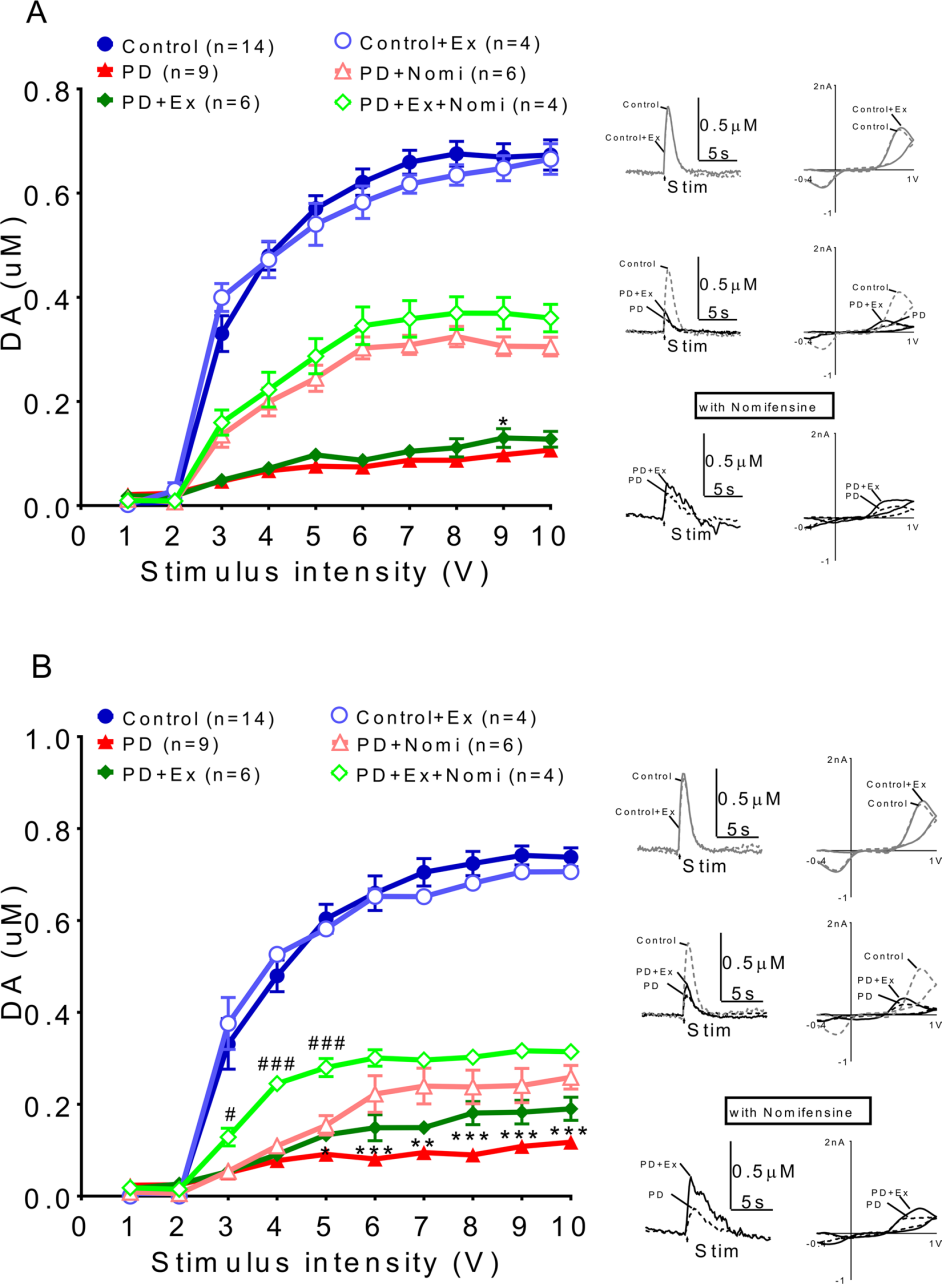
Analysis of dopamine release patterns in PD and PD with exercise animals. (**A**) Tonic release was very low after the 6-OHDA lesion and the signal could be enhanced by adding nomifensine (a DAT inhibitor). (**B**) Marked suppression of phasic DA release was seen in 6-OHDA lesioned animals compared to sham (sham, blue solid circle). Enhancement of release could be found after nomifensine infusion especially in the exercise group (green open diamond) compared with 6-OHDA lesioned only animals (pink open triangle).

By using fast cycle voltammetry (FSCV), we analyzed the dopamine release dynamics in PD and PD with exercise animals (Fig. 7). Tonic release (Fig. 7A) and phasic DA release (Fig. 7B) evoked by various stimulation intensities were very low after the 6-OHDA lesion and we used nomifensine, a DAT inhibitor, to augment the signal in order to confirm the dopamine signal. The enhancement of DA release could be found after nomifensine infusion particularly in exercise group (green open diamond), compared to 6-OHDA lesioned only animals (pink open triangle).

**Figure 8:**
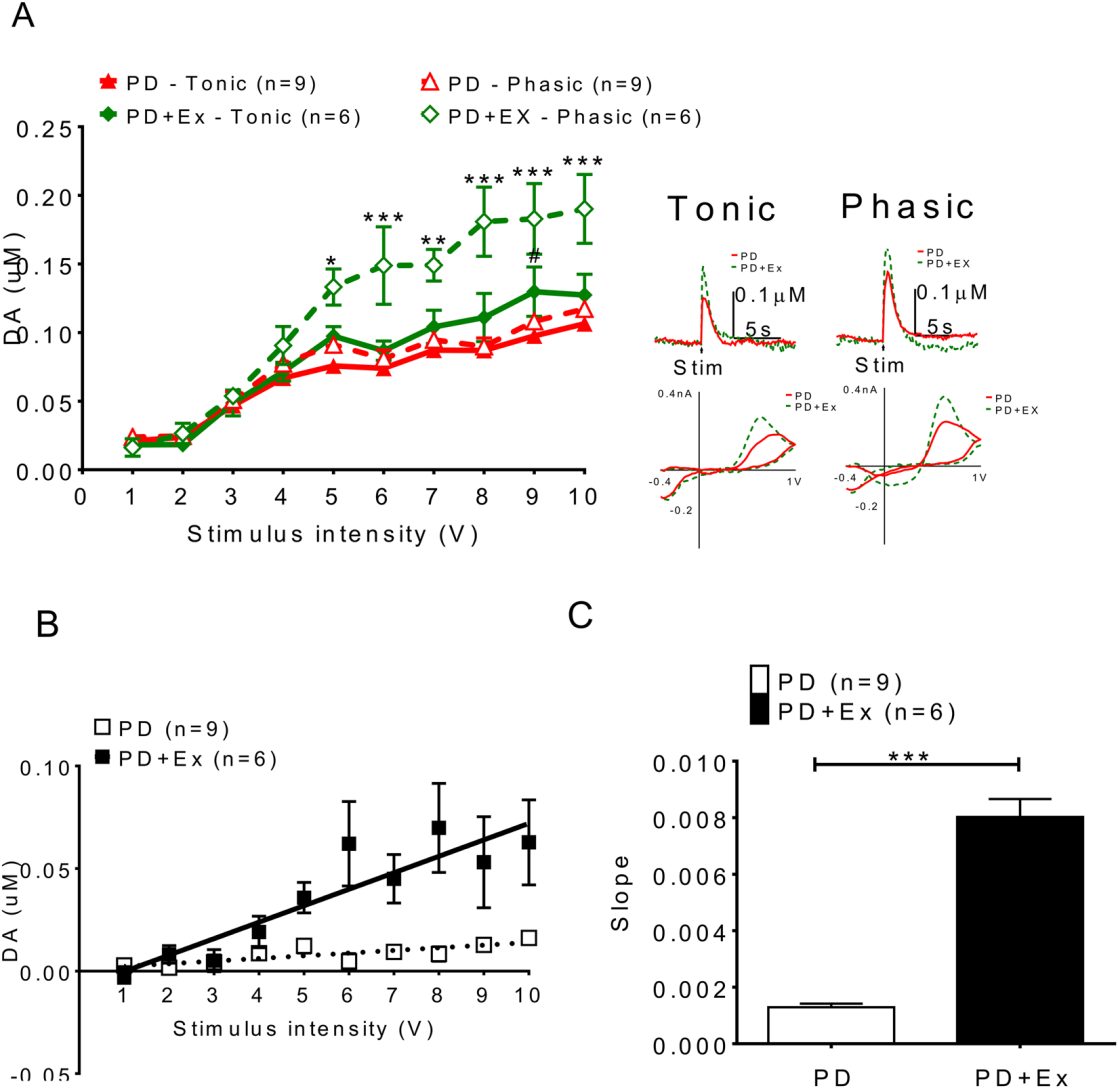
The deficits of dopamine release probability could be improved after exercise in PD animals. Dopamine release probability measured by using I/O curves generated by evoked DA responses under different stimulation intensities, which indicated that DA release on the 6-OHDA lesioned side could be improved by exercise not only with tonic release (solid diamond vs solid triangle) but also with phasic release (open diamond vs. open triangle). (**A**) Dopamine release increased in the exercise group and this increment became larger after nomifensine infusion. The dopamine release probability was calculated by different dopamine release concentrations between phasic (10p/25 Hz) and tonic (1p/25 Hz) different stimulation intensities, ([DA] d = [DA]10P-[DA]1P), (**B**) Linear regression of concentration difference under stimulation intensities 1~10 V. (**C**) The differences between the phasic and tonic release, referred as releasing probability (slope) increased in exercise animal comparing with those lesioned only. *which indicated a significant difference (p < 0.05) between the PD and PD with exercise animals in Phasic release. #which indicated significant difference (p < 0.05) between the PD and PD with exercise animals in Tonic release.

The release signals evoked by different situation intensities (from 1 to 10 volts) referred as input/output (I/O) curves were plotted, which indicated that DA release on the 6-OHDA lesion side could be improved by exercise not only for tonic release (PD: solid triangle vs. PD with exercise: solid diamond) but also for phasic release (PD: open triangle vs. PD with exercise: open diamond) (Fig. 8A). This increment in dopamine release became even more significant after nomifensine infusion.

The release probability measured with the FSCV signal has been documented previously^22^. Three voltammetric signals were obtained and averaged at each recording site using a single pulse (tonic) and 10 pulses (phasic) stimuli delivered at 25 Hz. The dopamine release probabilities were calculated by different dopamine release concentrations between phasic (10p/25 Hz) and tonic (1p/25 Hz) under different stimulation intensities, ([DA] _d_ = [DA]_10P_-[DA]_1P_). The data were fit to a linear regression model (y = mx + b; Prism 5.02; GraphPad, San Diego, CA, USA), where the slope m represents the relative change in DA concentration per pulse. The exercise group had a higher release probability than those in 6-OHDA lesion only animals (Fig. 8B), as indicated by the differences in the slope (in Fig. 8C).

### Dopamine reuptake rate in each group was analyzed further to determine the kinetic effect of exercise on dopaminergic release

**Figure 9:**
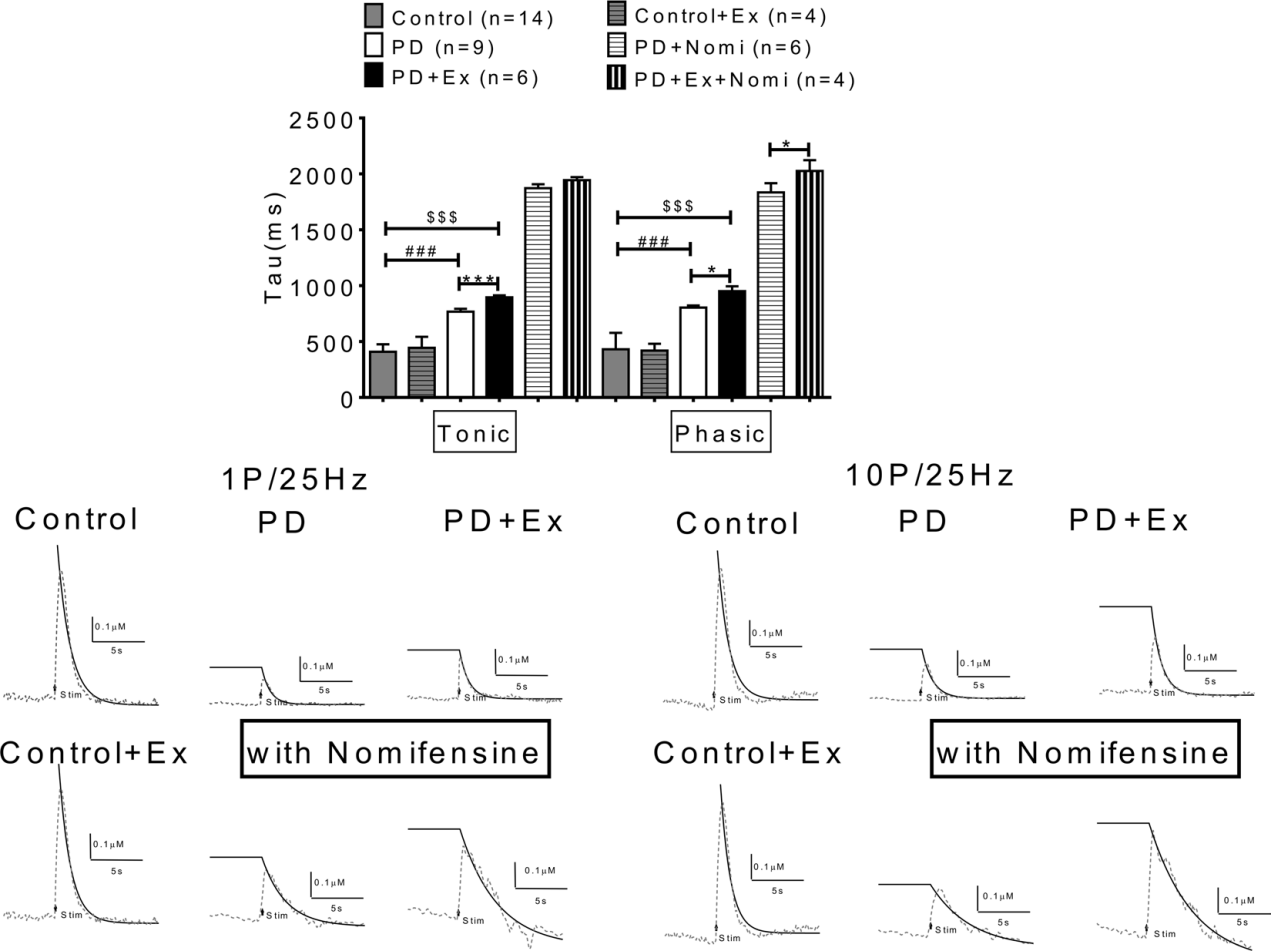
Dopamine reuptake were delayed by exercise. The kinetics of dopamine signals were determined by measuring the decay rate constant (τ) of evoked dopaminergic signals in brain slices in each group. The dopamine reuptake rate (tau) was lower in the exercise group; the tau value not only for tonic release but also for phasic release which was higher in PD animals. Nomifensine application did not affect the reuptake rate in the tonic phase but prolonged the reuptake rate of phasic release in the PD with exercise group.

The kinetics of the dopamine signal evoked by intrastriatal stimulation was studied by monitoring the cyclic voltammetry signal for 1 s before and 5 s after intrastriatal stimulation at a sampling rate of once every 100 ms (10 Hz). We then calculated the decay of the dopamine signal by normalizing post peak dopamine measurements to the peak dopamine measured. A decay constant was then determined as described in Methods, and the constant −k is the decay rate for exponential decay of the dopamine signal (Fig. 9). The dopamine reuptake rate was lower in the exercise group; thus in PD with exercise the tau value not only for tonic release but also for phasic release was higher than in PD animals. Nomifensine application did not affect the reuptake rate in tonic but prolonged the reuptake rate for phasic release in the PD with exercise group. Thus, our FSCV data indicate that exercise improves DA transmission in PD animals via dynamic effects in dopamine release enhancement as well as in delaying dopamine clearance rate.

Correlative changes in the nigrostriatal DA input evaluated by TH immunocytochemistry are shown in Supplementary Figure 1. Immunohistochemical staining of tyrosine hydroxylase (green) and NeuN (red) in the striatum after 4-weeks shows that severe depletions of TH were found in PD animals while an increment in TH stainings were seen in the striatum in PD with exercise animals. The quantifications of TH staining density in each group. The TH density on the lesioned side was higher in PD with exercise animals compared with PD only animals, although the TH density in the PD with exercise group was still lower than in control animals.

## Discussion

The typical PD triad: rigidity, resting tremor and bradykinesia, has been related to the extent of dopaminergic pathway loss. In addition to this classical triad, the issue of gait disturbances in PD has attracted attention because falls and imbalance may result in severe injury and affects the life quality^23,24^. Typical gait disorders in PD include stooped posture, festination, flexed knees, narrow base, reduced arm-swing, turning difficulty, and freezing of gait (FOG)^25^.

By using rats with unilateral 6-OHDA lesions that exhibit motor asymmetries and shuffling gait patterns during locomotion that resemble the key features of human parkinsonian gait^23,26^, we characterized gait impairment and compensation in a PD model for further translating research for novel treatments^27,28^. In this study, we focused on the connections between motor kinetic deficits, gait disturbances, corticostriatal plasticity and dopaminergic transmission impairments in PD rats, which were ameliorated by exercise.

Exercise improves the motoric deficits in PD. Our data revealed that the motor deficits including walking speed (Fig. 1E) and the rotarod test (Fig. 1D) were improved gradually after exercise, which indicated that exercise improves kinematics and motor coordination in PD animals, findings which are in accord with previous publications^29,30^.

Although apomorphine-induced rotations in both PD and PD with exercise animals, which was used as the behavioral confirmation of lesioning in both groups (Fig. 1A), were initially similar, the threshold dosage for apomorphine-induced rotation was higher in PD with exercise (Fig. 1G and H), and the dose-dependent linear regression line for apomorphine-induced rotation in the PD with exercise group was shifted to right (Fig. 1I). Since apomorphine-induced rotation depends on DA receptor supersensitivity, this suggests supersensitivity is reduced in the exercise group which fits well with our DA release and reuptake data.

Analyzing temporal–spatial gait parameters allows quantification of the gait cycle of individuals or animals^31^. The definition of these parameters is shown in Table 2. PD patients have a shorter stride length, slower velocity, and more unpredictable fluctuations between consecutive strides compared to healthy adults^32,33^. The sequence of footprints was used here to determine spatial gait parameters such as: the base of support (BOS), step length, stride length, and foot angle; dynamic limb function. Subtle temporal changes in a gait pattern such as natural walking speed, specific kinematic parameters, and exact gait cycle can also been analyzed^28^.

**Table 2:** Basic parameters of gait and their definitions.

Gait parameter	Definition
Stride time	Time between two successive ground contacts of same feet.
Step time	Time between two successive ground contacts of the opposite feet.
Stride length	Distance covered between two successive ground contacts of the same foot
Step length	The distance form initial contact of one limb to the initial contact of the opposite limb.
Base of support	The distance between initial contact of one limb and the initial contact of the opposite limb
Foot angle	The angle of external rotation of the foot
Print length	The distance from the third toe to the heel
The intermediary toe spread	The distance from the second to the fourth toe
Toe spread	The distance from the first to the fifth toe
Stance time	The time of contact of the foot with the floor
Swing time	The time of the rear foot leaving the floor
Double support	The time at which both feet are in contact with the floor at the same time

Fixed speed treadmill training exercise improved the gait deficit in PD and the data is summarized in Table 1, which indicates that exercise improved motor deficits in walking speed and knee angle variation. The majority of spatiotemporal indices of gait in PD were improved by exercise.

Axial rigidity, interlimb incoordination and asymmetries in PD results from impaired sensorimotor integration, inability to switch between sensory modalities and lack of compensatory stepping. All of these deficits contribute to high incidence of falls in patients with Parkinson’s disease^24^. In addition, temporal processing^34^ and internal rhythmic timing are disrupted in PD with gait deficits^35,36^ A growing body of evidence has indicated that the basal ganglia- supplementary motor area - premotor cortex(BG–SMA–PMC) network is directly involved in rhythm perception^37,38^. Dorsal striatum (caudate and putamen) of the BG serves the most crucial role in generating the internal pacing required for time estimation^39^; this internal pacing is controlled via the D2 receptors in the striatum^40,41^.

Fixed speed treadmill or regular physical exercise training may provide rhythmical activities, induce neural circuit rewiring and plasticity, and improve the gait deficits^36,42^.

In addition to the spatiotemporal analysis of gait, ankle joint trajectories were used to evaluate four specific stages during locomotion, which were previously developed to measure motor recovery after peripheral nerve injury^43^. In our investigation, the ankle joint angle of PD animals was decreased initially at the toe contact stage and then significantly decreased at the mid standing and pres-swing phases compared with sham animals (Fig. 5). This decreased joint angle at toe contact stage until the pre-swing stage could explain why PD rats require more contact area of the paw to increase balance and stability during locomotion^28^. The ankle angle reversed gradually after training in the PD with exercise group.

The improvement of corticostriatal pathway parameters were shown in PD animals with exercise. The PD animals without exercise had very short retention times in rotarod tests (Fig. 1C and D). Motor and gait control relates to the corticostriatal pathway and basal ganglia function^44^. Neuroplasticity with respect to “motor learning” may be composed various mechanisms such as synaptogenesis, neurogenesis, and neuronal sprouting to potentiate synaptic strength^45^. Plasticity in the corticostriatal pathway may have important roles not only in gait control^46^ but also motor learning^47^. Synaptic plasticity and rewiring in M1 cortex may be important in learning and memory, because basal ganglia receive glutamatergic inputs from the primary motor cortex (M1) which is important for motor control and the acquisition of motor skills^48^. Moreover, dopaminergic signaling within M1 modulates synaptic plasticity, which is important for optimizing motor learning^49^. The distinct roles for D1 and D2 dopamine receptor signaling in regulating spine dynamics and functional plasticity in M1 have been delineated^49,50^, and these are disrupted by dopamine depletion. Dopaminergic afferents to the M1 cortex originate from two different systems: directly innervated from the mesolimbic system and indirectly innervated from the nigrostriatal system via basal ganglia. Thus, abnormal spine turnover in the motor cortex may contribute to motor deficits observed in PD^51^.

In our study corticostriatal pathway plasticity in PD animals could not be induced, whereas bi-directional plasticity could be induced in the PD with exercise group (Fig. 6C) This partial restoration may result from exercise augmenting neurotransmitters (e.g. dopamine) and trophic factor synthesis^44,52^, which both promotes neuroplasticity and decreases neural apoptosis.

The dynamic and kinetic effects of exercise on dopaminergic transmission in PD animals. Our FSCV results indicated that dopamine release signals were very low in both PD and PD with exercise animals (Fig. 7). However, exercise could improve dopaminergic transmission in the exercise PD group (Fig. 8A). The dopamine release concentration difference between tonic and phasic release, referred as release probability, increased significantly in the PD with exercise group (Fig. 8B and C). On the other hand, the released DA concentration in the synaptic cleft in the PD with exercise group may also be related to delayed clearance, as shown in Fig. 9. This data is compatible with previous reports that indicated that exercise may decrease DAT number or expression^53^. That dopaminergic neuron loss is ameliorated by exercise like treadmill training has also been shown^54^.

Exercise enhanced recovery after nigrostriatal DA injury may result from a number of factors: physical exercise activates antioxidant enzymes and reduces chronic oxidative stress; exercise stimulates mitochondrial biogenesis, and there is up-regulation of mitophagy in PD patients^44^. Exercise also stimulates trophic factor synthesis (BDNF, GDNF, FGF-2, IGF-1, among others), which promotes neuroplasticity and decreases neural apoptosis^44^, and which alters dopaminergic neurotransmission^55,56^. Exercise may exert neuroprotective effects or enhance the neuronal survival by increasing NTF availability and elevating intracellular defenses against ROS after 6-OHDA. This in turn increases the capacity of DA neurons to deliver transmitter and reduces vulnerability of those neurons^57,58^.

We also found that neuroinflammation after 6-OHDA is ameliorated by 4 weeks of treadmill exercise (supplementary data Fig. 3). To determine neuroinflammatory reactions after 6-OHDA we performed CD11b staining for microglial activation and GFAP stains for glial activation. To further determine the activation of microglia, we quantitated the subtypes of morphology of microglia. Fractal dimensions (Df) on three slides (40×) of each group were measured by Image J using the Fraclac analysis method^59,60^. The ramified microglia were found to have higher Df values than the activated microglia. Our data indicates that microglial activation, induced by 6 –OHDA, can be ameliorated by exercise (supplementary data Fig. 3B and E). The same situation is found by using GFAP staining to evaluate glial activation, while the marked gliosis induced by 6-OHDA lesioning is ameliorated after exercise on lesioned side striatum (supplementary data Fig. 3D and F). Moreover, there is much literature on glial activation and neuroinflammatory changes after 6-OHDA lesions and how markers for these are reversed by exercise. Real *et al*.^61^ showed glial activation evaluated by [11 C]PBR28 PET and GFAP, which were reversed by exercise. Similarly, Dutra *et al*.^62^ showed restoration of GFAP changes in 6-OHDA-lesioned rats after exercise. In terms of neuroinflammatory markers, several studies have shown upregulation in the 6-OHDA model which were reversed by exercise. Real *et al*.^63^ showed that microglial inflammatory markers were upregulated after the 6-OHDA lesions in rats and these were reversed by exercise. In this same paper, markers for glial activation (GFAP) and reactive oxygen species (iNOS, DHE) were upregulated in sedentary rats after the 6-OHDA lesion but were normalized in treadmill exercising animals, similar to treadmill exercise in our study.

Our results are also compatible with previous data showing that physical exercise (PE) constitutes an effective intervention in other neurodegenerative diseases and attenuates disease progression^64–66^. The mechanisms contributing to this phenomena may not only derive from peripheral effects of acute PE including increasing cardiac output and cerebral blood flow^67^, but also derive directly from CNS effects on several neurobiological mechanisms including increases in angiogenesis, neurogenesis, synaptogenesis, and neurotransmitter synthesis in different cerebral areas involved in cognition and mobility in PD^68,69^. Importantly, our data also support previous reports that exercise-induced benefits on brain health (ie, blood flow, trophic factors, and the immune system) might help to create an optimum milieu for neuroplasticity in the injured brain^53^. We have summarized the potential mechanisms associated with our data in Table 3.

**Table 3:** The effect and mechanism of exercise in PD animal.

Mechanism	Result	Reference
*Neurogenesis*	1. Many of the molecules that are increased by running, including serotonin, insulin-like growth factor, and BdNF, have been causally linked to running- enhanced neurogenesis.	^82–86^
	2. Proliferation of astrocytes and activation of microglia	
	3. HIF 1-alpha expression: increasing fuel availability glucose transporters (GLUT-1 and GLUT-3) and glycolytic pathway enzymes and also promote neurogenesis, synaptogenesis, and angiogenesis	^87^
	4. Physical exercise-induced changes in the hippocampus *in vivo* and confirms possibility of angiogenesis/neurogenesis underlying plasticity processes.	^14^
*Neuroplasticity*	1. Skill aerobic exercise (SAE) elevated expression of both presynaptic (Synaptophysin) and postsynaptic (PSD-95) proteins.	^53^
	2. Intensive treadmill running can reverse the loss of dendritic spines on striatal MSNs [50] Dendritic spine density in the B.G	
	3. Exercise may alleviate brain inflammation-induced learning impairment: The long-lasting effect of exercise on LTP through enhancement of the expressions regarding BDNF, TrkB, and p-CREB. Treadmill exercise and wheel exercise exerted similar effects on these factors.	^88^
	4. MPTP-exercise group increases expression of synaptophysin, PSD-95, TH, and dendritic spine in nigrostriatal dopaminergic neurons and fibers than MPTP treated group	^89^
*Neuroprotection*	1. Aerobic exercise regulates Rho/cofilin pathways to rescue synaptic loss	^90^
	2. Exercise activate the signaling pathways underlying brain protection.	^91^
	3. Exercise increased availability of NTFs, which in turn can promote mitochondrial energy production, antioxidant defense, synaptogenesis, reduced inflammation, angiogenesis, and other processes that suppress apoptosis.	^92^
	4. By promoting synaptic plasticity and neurogenesis in the hippocampus, BDNF mediates exercise induced improvements in cognitive function and neuroprotection	^93,94^
*Neurotransmission*	1. Enhacing DA transmission	^53^
	i. Enhance vesicular release of dopamine,	
	ii. Increase of synaptic occupancy, and	
	iii. Decrease of dopamine clearance through reduced DAT expression.	
	2. Effect of Glutamate transmission	
	i. Exercise alters the AMPA receptor subunit GluA2 expression, particularly localized to indirect DA-D2R containing MSNs	^95^
	ii. Exercise reduces synaptic excitability and postexcitatory synaptic potentials	
	iii. Exercise reduces the presynaptic storage of glutamate.	^96^
	iv. Reduces aberrant glutamatergic drive to restore cortico-striatal circuit function	
*Altering the BBB*	Increases the availability of biomoleculesto enhance synapse formation and ameliorate the inflammation	^97^
*Maintenance of cellular homeostasis*	Physical exercise directly influences the responsiveness of CNS circuits involved in energy homeostasis.	^98,99^
*Cerebral blood circulation*	Aerobic exercise (AE) enhanced circulatory and respiratory efficiency that improves the body’s use of oxygen, and increase in the density of capillaries in the brain’s motor regions.	^100,101^
	SAE resulted in greater increases in regional cerebral blood flow (rCBF) and prelimbic cortical activation	

## Material and Methods

### Animals

Male adult Sprague-Dawley rats (280–300 g) were purchased from the National Laboratory Animal Center, Taipei, Taiwan, R.O.C. All rats were housed in an environment of 12 h light/dark cycle, temperature of 25 ± 2 °C, 55% humidity, 2–3 animals per cage, and ad libitum standard diet and water at the National Defense Medical Center’s Animal Center, which is accredited by the Association for Assessment and Accreditation of Laboratory Animal Care International (AAALAC) International. Their care was in accordance with institutional and international standards (Principles of Laboratory Animal Care, NIH). The experimental protocol was approved by the Institutional Animal Care and Use Committee (IACUC; protocol number 16-258) of the National Defense Medical Center, Taiwan, ROC.

### 6-OHDA-induced hemiparkinsonism

For the 6-OHDA lesion or sham operations, rats were anesthetized with intraperitoneal Zoletil 50 (50 mg/kg) plus Rompun (2 mg/kg) and placed into a stereotactic apparatus (Stoelting, IL, USA). They were lesioned in the right medial forebrain bundle (MFB, AP -4.3 mm, ML + 1.6 mm, DV-8.2 mm) with 4 μg/μl of 6-hydroxydopamine (6-OHDA alone freebase weight: 169.18; and we used the HCl form: 205.638, dissolved in 0.02% ascorbic acid, Sigma Chemical Co., USA). The 6-OHDA solution (total volume of 4 μl) was injected over 8 min period at an injection rate of 0.5 μl/min and the needle left in place for additional 5 min before retraction. In sham groups, 0.02% ascorbic saline (total volume of 4 μl) was injected into right MFB.

It should be noted that although actual DA cell loss after 6-OHDA bundle lesions may take several weeks, a profound loss of the DA phenotype occurs much more rapidly. Contralateral turning after low dose of apomorphine, reflecting receptor deviation supersensitivity, is readily seen one week after a successful 6-OHDA bundle lesion^19^. With 6-OHDA infrastructural lesion, DA loss occurs much more gradually, over several weeks, and the magnitude of loss is much less than with bundle lesions. This was the rationale for the bundle lesion techniques used here (see below).

### Experimental design and Exercise treatment

The rats in the PD group received the 6-OHDA injection to induce hemiparkinsonism on day 1. The rats were pre-trained before surgery to make sure that they can walk on the straight treadmill (Ugo Basile #47300). One week after the 6-OHDA lesion, the rats in the exercise groups (N = 6) were forced to run on a motorized treadmill (11 m/min for 30 min each day, consecutive 5 days/week) for 4 weeks. The rats in the non-exercise groups (N = 6) remained on the treadmill for the same duration of time without running. The behavioral tests (apomorphine-induced rotation, rotarod, and gait analysis) were performed before and every week after the 6-OHDA lesion for 5 weeks. The rats were sacrificed after all the behavioral tests.

### Apomorphine-induced rotation

The effectiveness of the MFB lesion was confirmed by an apomorphine-induced rotational test every week following the 6-OHDA lesion. The dopamine receptors become supersensitive after 6-OHDA-induced DA denervation. Apomorphine, a dopamine receptor agonist, causes an asymmetrical increase on that side relative to the non-affected side and resulting in contralateral turning^70^. Apomorphine-induced rotation was observed for 60 min after a subcutaneous injection of 0.05 mg/kg apomorphine (Sigma–Aldrich, USA) dissolved in 0.1% ascorbic acid solution in a rotation bowl (38 cm wide at top and 20 cm deep) and the number of ipsilateral and contralateral turns was quantified. Rats that failed to demonstrate apomorphine-induced contralateral rotation behaviors, suggesting an incomplete lesion, were excluded from further analysis. Total 31 of male SD rats were used and 15 of them were treated with 6-OHDA while 3 excluded after the lesion assessment.

### Rotarod test

Motor coordination and balance was assessed by an automated 4-lane rotarod unit (Ugo Basile, Italy) every week after 6-OHDA lesion. The rats were pre-trained for 2 days in order to reach a stable performance. The rats were mounted on the rotarod (18 RPM) and the latency to fall from the rod was automatically recorded. Motor balance was assessed before and after neurotoxin injection at four consecutive times, each lasting 720 s. Values were expressed as retention time on the rotarod in the four test trials.

### Gait analysis

For gait analysis, a walking track equipped with a video-based system was adapted from the design by Hsieh *et al*.^8,28^. The definitions of spatiotemporal parameters of gait are shown on Table 2. The walking track apparatus consisted of a plexiglass chamber that was 80 (l) x 6 (w) x 12 (h) cm. A mirror was placed at an angle of 45° to the walking track to reflect the image of the rat’s paw for observation with a digital camera^28,71^. Initially, the rats were allowed to walk freely on the track for acclimation. For each rat, three satisfactory walks of at last 4 steps without pause were recorded for analyses. The walking speed, step length, base of support, stride length, foot angle, print length, intermediary toe spread, toe spread, stance time, swing time, and double support were determined using Matlab software (MatWorks, version 7.6., R2008a).

### Electrophysiological recordings and measurement of striatal synaptic plasticity

Extracellular recordings of striatal population spikes were performed in the dorsal striatum using previously described techniques^72^. Extracellular population spike recordings were used rather than whole-cell voltage clamp recordings because population spikes reflect the simultaneous activity of a large number striatal MSNs. To isolate glutamate-driven population spikes from GABAA-mediated currents with a similar time course^73^, the aCSF contained the GABAA receptor/Cl channel blocker picrotoxin (100 μM). Electrical stimulation was performed using bipolar tungsten stimulating electrodes (Frederick Haer, Bowdoin, ME, USA) placed on the tissue either close to (<100 μm) the intrastriatal recording electrode or 1–2 mm above the corpus callosum, at the border of primary motor and somatosensory cortices. Baseline population spike responses were elicited using single 0.1 ms pulses (10–30 V) delivered through a stimulating electrode at a frequency of 0.033 Hz. The stimulus intensity was then adjusted to elicit a response that was 40–50% of the maximum and the stimulator was left at this setting for the remainder of the experiment. Data were acquired and stored on a personal computer via an A/D board (National Instruments PCI 6024E, Austin, TX, USA) using a Windows-based software package (WCP, courtesy of Dr. John Dempster, University of Strathclyde, Glasgow, UK; http://spider.science.strath.ac.uk/PhysPharm/showPage.php?pageName = software_ses). Population spike amplitudes were analyzed off-line using the same software. Comparisons were made between averages of at least 10 responses obtained during the baseline control (pre-high frequency stimulation (HFS)) period or at a fixed time (usually 60 min) after application of HFS as described below.

After stable baseline population spikes were measured, the synaptic plasticity of the corticostriatal pathway was assessed by examining the effects of high-frequency trains (HFS) applied through the stimulating electrode placed in the cortex. The high-frequency stimulus protocol^74,75^ consisted of a total of 400 pulses delivered in four trains of 1 s duration, separated by 10 s intervals, at 100 Hz (HFS-400). Population spikes were then monitored for at least 1 h after delivery of the high-frequency trains. The effects of PD on these forms of striatal synaptic plasticity have not been previously evaluated.

### Fast scan cyclic voltammetry and dopamine measurement in striatal slices

Fast-scan cyclic voltammetry (FSCV) was performed using carbon fiber electrodes as described previously^76,77^. Carbon fibers (7 μm diameter, Goodfellow Corp., Devon, PA, USA) were aspirated into glass micropipettes, which were then pulled using a multistage patch pipette program on a Sutter P-97 electrode puller. Carbon fibers, prepared as previous described^78^, were trimmed to allow ~ 20–50 μm to protrude from the glass capillary, and then sealed in the tip of the pipette by passing it quickly over a flame. Pipettes containing the carbon fiber were filled with a solution of 4M K-acetate/150 mM KCl and attached to the head stage of a patch clamp amplifier (HEKA EVA-8, HEKA Instruments Inc., Southboro, MA, USA). Pipettes were then inserted ~75–100 μm into the dorsal striatal brain slice and positioned between the separated tips of a bipolar stimulating electrode (FHC Inc., Bowdoin, ME, USA) using a stereo microscope. Voltammetric scans, stimulus wave form generation and timing, and data collection were performed using A/D boards (PCI 6052E and PCI-6711E, National Instruments, Austin, TX, USA) and custom LabView-based software (TarHeel CV, courtesy of Drs. Joseph Cheer and Michael Heien, University of North Carolina). Voltammetric scans from 0.4 to 1.0 V and back were performed at 100–400 V/s (7 to 28 ms scan duration) at a frequency of 10 Hz.A 5 second background measurement (50 scans) was taken prior to electrical stimulation of the brain slice and subtracted from the voltammetric scan obtained at the signal peak immediately after electrical stimulation. This was used to generate a voltammogram (current vs. voltage plot) for each signal. Electrical stimulation consisting of a single 4 ms biphasic pulse, which did not overlap the voltammetric scans, was used to release dopamine. Constant current stimulus intensity was varied from 0.1 to 1 mA in order to construct input-output curves for each placement in the striatal slice. All signals matched those expected for the oxidation and reduction of dopamine^79^.

To survey kinetics of the dopamine signal, decay of the signal evoked by intrastriatal stimulation was determined by normalizing postpeak dopamine measurements to the peak of measured dopamine. Time constants (τ) for the decay phase of each dopamine signal were obtained by fitting a single exponential function using a least-squares minimization algorithm: Y(t) = A-t/τ, where A = peak signal amplitude (nA), t = time (ms), and Y = signal amplitude at any given t. Initial comparisons of the sum of squares (F-test) between single and double exponential functions (Prism v. 6.01) confirmed that the decay phase was best fit by a single exponential in all cases. It has been demonstrated that the first-order rate constant (k, or 1/τ) obtained using this approach provides an index of the efficiency (Vmax/Km) of dopamine clearance mediated via the dopamine transporter at low dopamine concentrations^80^, when A is the peak dopamine signal at time 0 and the constant −k is the rate for exponential decay of the dopamine signal. ANOVAs were performed between all groups for the decay rate constant (−k)^55,81^.

The primary advantage to FSCV over chronoamperometry or amperometry is that the background-subtracted voltammogram (i.e. current vs. voltage plot) describes the entire kinetic process of electron transfer. Thus, well-defined peak oxidation and reduction currents can be used to distinguish among a number of different compounds (see Figure below from (Heien *et al*., 2004)). For example, dopamine (DA) is readily distinguishable from serotonin (5-HT) by both the width of the oxidation peak and the location of the reduction peak (−0.2 V for DA vs 0.1 V for 5-HT). Either of these compounds is easily resolved from ascorbate, which has slow, nearly irreversible oxidation. Likewise, electron transfer kinetics are slower with DOPAC, the primary acid metabolite of DA. Unfortunately, DA and NE show identical voltammograms, and cannot easily be resolved. In order to distinguish between these two catecholamines, it is best to select brain areas with minimal overlap (or where one species contributes predominately over the other as we have done here using the dorsal striatum site; see (Herr *et al*., 2012)). This, combined with pharmacological strategies or selective lesions of DA or NE pathways, can be used. In our study, for example, the MFB technique is quite selective for the nigrostriatal DA pathway. Moreover, the DAT blocker, infusion of nomifensine was used to enhance the DA signal here.

### Statistics

(a) Exercise effects on the motor deficit in PD rats and spatiotemporal analysis of gait in sedentary and exercise groups:

A one-way ANOVA and a Bonferroni post hoc test were used to determine apomorphine induced rotation (post lesion 1 week) and data from 2nd to 5th weeks post-lesion were averaged for rotarod, walking speed, spatial supporting parameters (base of support, step length and stride length), supporting parameters of paw and hind limb (print length, toe spread length, intermediate toe spread and foot angle), temporal parameters of gait (stance time, swing phase and double support) and kinetic parameters (toe contact, mid-stance, pre-swing and mid- swing phase).

(b) The effect of exercise on cortico-striatal pathway plasticity in PD rats and the effect of exercise on dopaminergic transmission:

Statistical analyses of data for the dopamine release input/output curves, cortico-striatal pathway input/output curves and synaptic plasticity were performed using a two-way analysis of variance (ANOVA) followed by a Bonferroni post hoc test for multiple comparisons. Statistical analyses of data for dopamine reuptake and slope of differences between the phasic and tonic release were performed using an unpaired t-test.

All statistical tests were two-tailed and were performed using GraphPad Prism 5.02 (GraphPad Scientific, San Diego, CA, USA). A p-value < 0.05 using a two-tailed test was considered significant.

### Significance Statement

This paper describes changes in gait parameters in the hemi-parkinsonian rat model and how such changes are ameliorated by exercise. Correlative changes in cortico-striatal plasticity as well as in dynamics of dopamine release and availability are also presented.

## Electronic supplementary material

Supplementary data Figure. 1

Supplementary data Figure. 2-1

Supplementary data Figure. 2-2

Supplementary data Figure. 2-3

Supplementary data Figure. 3
