# Supplementary material for: Exercise Ameliorates Motor Deficits and Improves Dopaminergic Functions in the Rat Hemi-Parkinson’s Model
Source: Sci Rep. 2018 Mar 5;8:3973. doi: 10.1038/s41598-018-22462-y (PMC5838260; doi:10.1038/s41598-018-22462-y)

**Exercise Ameliorates Motor Deficits and Improves Dopaminergic Functions in the Rat Hemi-Parkinson's Model**

Yuan-Hao Chen^1*^ MD, PhD, Tung-Tai Kuo^2^ MA, Jen -Hsin Kao^1^ PhD, Eagle Yi-Kung Huang^3^ PhD, Tsung-Hsun Hsieh^4^ PhD, Yu-Ching Chou^5^ PhD, Barry J Hoffer^6, 7^ MD, PhD

^1^Department of Neurological Surgery, Tri-Service General Hospital, National Defense Medical Center, Taipei, Taiwan, R.O.C.

^2^ Graduate Institute of Computer and Communication Engineering, National Taipei University of Technology, Taipei, Taiwan, R.O.C.

^3^ Department of Pharmacology, National Defense Medical Center, Taipei, Taiwan, R.O.C

^4^ Department of Physical Therapy and Graduate Institute of Rehabilitation Science, Chang Gung University, Taoyuan, Taiwan

^5^ School of Public Health, National Defense Medical Center, Taipei, Taiwan. , R.O.C

^6^ Graduate Program on Neuroregeneration, Taipei Medical University, Taipei, Taiwan.

^7^Department of Neurosurgery, Case Western Reserve University School of Medicine, Cleveland, Ohio, USA

*Corresponding author: Yuan-Hao Chen

E-mail: [chenyh178@gmail.com](mailto:chenyh178@gmail.com) (Y-HC)

**Supplementary data Figure.1**

Histological examination of the dopaminergic striatal system in PD and PD with exercise animals were surveyed. (A) Immunohistochemical staining of tyrosine hydroxylase (green) and NeuN (red) in the striatum after 4-weeks shows that severe depletions of TH were found in PD animals while (B) an increment in TH stainings were seen in the striatum in PD with exercise animals. (C) The quantifications of TH staining density in each group. The TH density on the lesioned side was higher in PD with exercise animals compared with PD only animals, although the TH density in the PD with exercise group was still lower than in control animals. (mean ± SEM; n = 12

for the Control group, n = 11 for the PD, n = 12 for the PD+Ex, one-way ANOVA, F = 73.89, followed by Bonferroni post test; *** p < 0.001; ** p < 0.01. Scale bar - 30 μm)


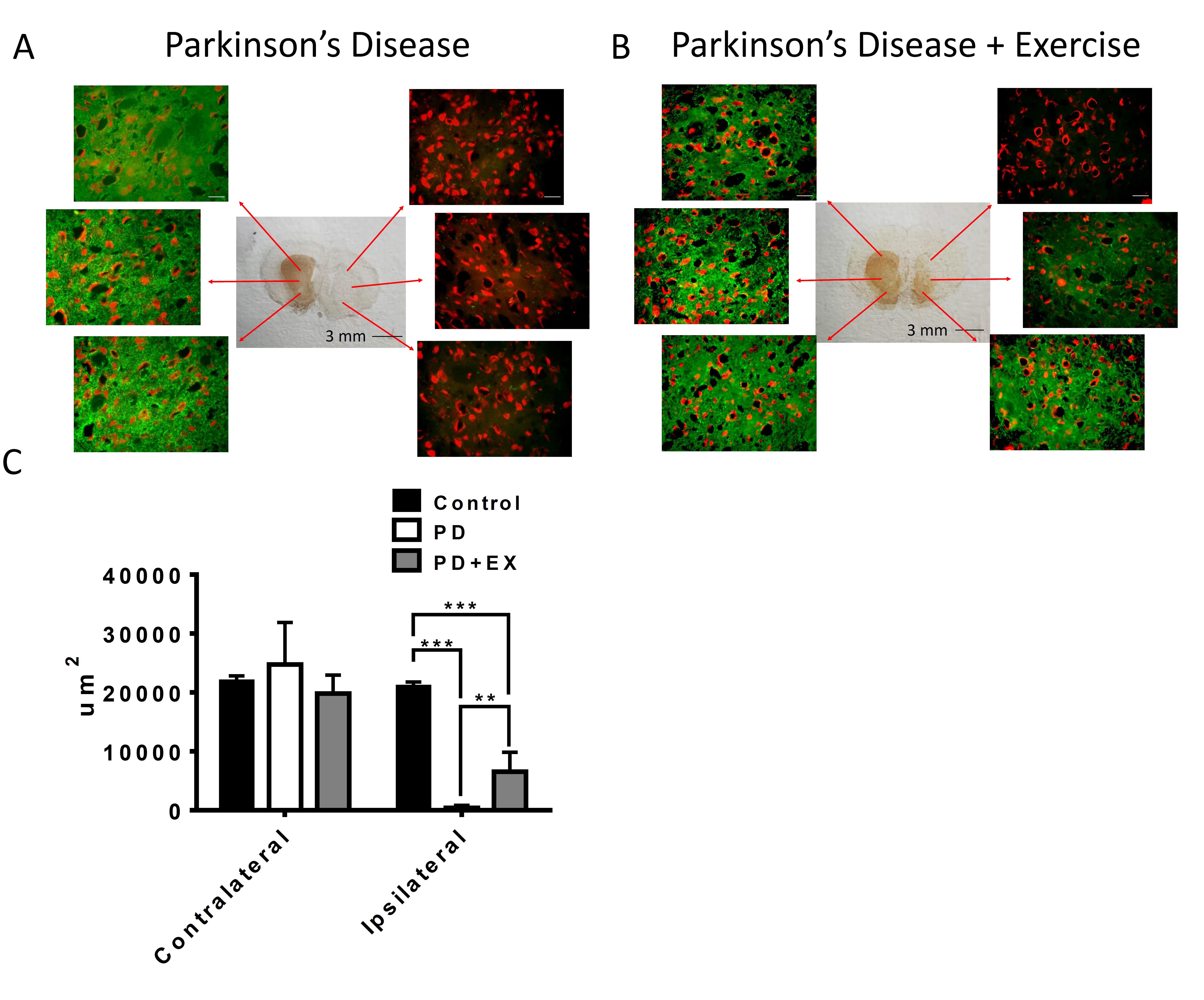

Supplement: Supplementary file 1 — Supplementary data Figure. 1 [file 41598_2018_22462_MOESM1_ESM.docx]
