# Supplementary material for: Exercise Ameliorates Motor Deficits and Improves Dopaminergic Functions in the Rat Hemi-Parkinson’s Model
Source: Sci Rep. 2018 Mar 5;8:3973. doi: 10.1038/s41598-018-22462-y (PMC5838260; doi:10.1038/s41598-018-22462-y)

**Exercise Ameliorates Motor Deficits and Improves Dopaminergic Functions in the Rat Hemi-Parkinson's Model**

Yuan-Hao Chen^1*^ MD, PhD, Tung-Tai Kuo^2^ MA, Jen -Hsin Kao^1^ PhD, Eagle Yi-Kung Huang^3^ PhD, Tsung-Hsun Hsieh^4^ PhD, Yu-Ching Chou^5^ PhD, Barry J Hoffer^6, 7^ MD, PhD

^1^Department of Neurological Surgery, Tri-Service General Hospital, National Defense Medical Center, Taipei, Taiwan, R.O.C.

^2^ Graduate Institute of Computer and Communication Engineering, National Taipei University of Technology, Taipei, Taiwan, R.O.C.

^3^ Department of Pharmacology, National Defense Medical Center, Taipei, Taiwan, R.O.C

^4^ Department of Physical Therapy and Graduate Institute of Rehabilitation Science, Chang Gung University, Taoyuan, Taiwan

^5^ School of Public Health, National Defense Medical Center, Taipei, Taiwan. , R.O.C

^6^ Graduate Program on Neuroregeneration, Taipei Medical University, Taipei, Taiwan.

^7^Department of Neurosurgery, Case Western Reserve University School of Medicine, Cleveland, Ohio, USA

*Corresponding author: Yuan-Hao Chen

E-mail: [chenyh178@gmail.com](mailto:chenyh178@gmail.com) (Y-HC)

**Supplementary data Figure.2-1**

The spatial support parameters between limbs of PD animal improved after exercise. (A) instep length (StepL) (Two-way ANOVA[F = 1.614, p = 0.1270] followed by Bonferroni post hoc test; *denotes p < 0.05, **denotes p < 0.01 PD vs. PD+Ex, #denotes p < 0.05 Sham vs. PD) (B) stride length (StideL) on the contralateral (healthy) side limb (right) of PD animals also revealed significant improvements after 3~ 4 weeks exercise (Two-way ANOVA[F = 2.183, p = 0.033] followed by Bonferroni post hoc test; *denotes p < 0.05, ***denotes p < 0.001 PD vs. PD+Ex, ##denotes p < 0.01 Sham vs. PD) and (C) The bar chart shows average post-lesion 2nd to 5th week’s data from each group. (“RStepL” One-way ANOVA[F = 2.61, p = 0.1276] followed by Bonferroni post hoc test; “RStrideL” One-way ANOVA[F = 8.399, p = 0.0108] followed by Bonferroni post hoc test; #denotes p < 0.05 Sham vs. PD)


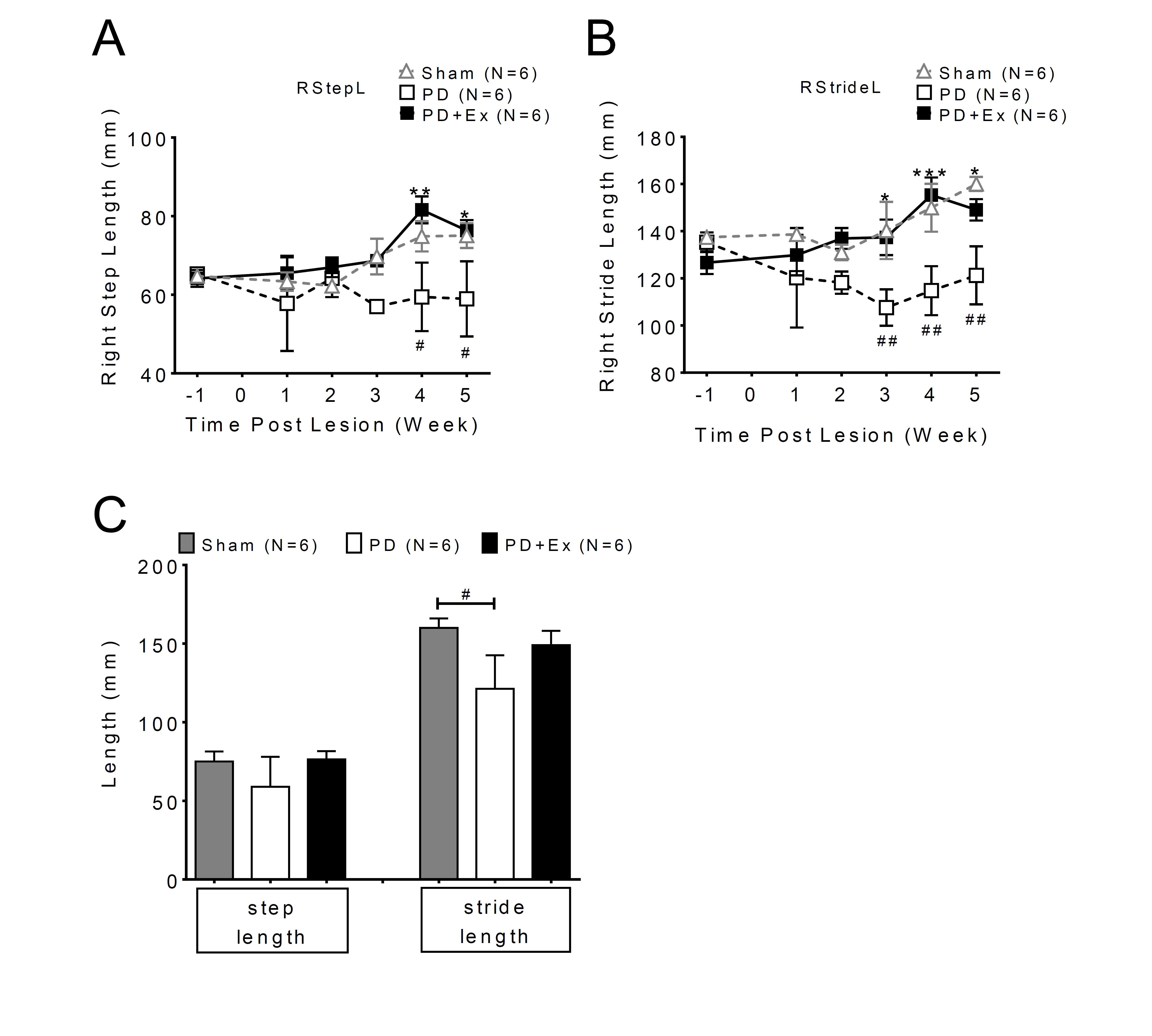

Supplement: Supplementary file 2 — Supplementary data Figure. 2-1 [file 41598_2018_22462_MOESM2_ESM.docx]
