# Supplementary material for: Exercise Ameliorates Motor Deficits and Improves Dopaminergic Functions in the Rat Hemi-Parkinson’s Model
Source: Sci Rep. 2018 Mar 5;8:3973. doi: 10.1038/s41598-018-22462-y (PMC5838260; doi:10.1038/s41598-018-22462-y)

**Exercise Ameliorates Motor Deficits and Improves Dopaminergic Functions in the Rat Hemi-Parkinson's Model**

Yuan-Hao Chen^1*^ MD, PhD, Tung-Tai Kuo^2^ MA, Jen -Hsin Kao^1^ PhD, Eagle Yi-Kung Huang^3^ PhD, Tsung-Hsun Hsieh^4^ PhD, Yu-Ching Chou^5^ PhD, Barry J Hoffer^6, 7^ MD, PhD

^1^Department of Neurological Surgery, Tri-Service General Hospital, National Defense Medical Center, Taipei, Taiwan, R.O.C.

^2^ Graduate Institute of Computer and Communication Engineering, National Taipei University of Technology, Taipei, Taiwan, R.O.C.

^3^ Department of Pharmacology, National Defense Medical Center, Taipei, Taiwan, R.O.C

^4^ Department of Physical Therapy and Graduate Institute of Rehabilitation Science, Chang Gung University, Taoyuan, Taiwan

^5^ School of Public Health, National Defense Medical Center, Taipei, Taiwan. , R.O.C

^6^ Graduate Program on Neuroregeneration, Taipei Medical University, Taipei, Taiwan.

^7^Department of Neurosurgery, Case Western Reserve University School of Medicine, Cleveland, Ohio, USA

*Corresponding author: Yuan-Hao Chen

E-mail: [chenyh178@gmail.com](mailto:chenyh178@gmail.com) (Y-HC)

**Supplementary data Figure.2-2**

The support parameters of paw and hind limb were also improved on the right side (healthy side of animals) in (A) print length (Two-way ANOVA[F = 1.326, p = 0.2403] followed by Bonferroni post hoc test; *denotes p < 0.05, **denotes p < 0.01 PD vs. PD+Ex, #denotes p < 0.05, ###denotes p < 0.001 Sham vs. PD), (B) toe spread length(Two-way ANOVA[F = 1.14, p = 0.3509] followed by Bonferroni post hoc test; #denotes p < 0.05, ##denotes p < 0.05, ###denotes p < 0.001 Sham vs. PD; $denotes p < 0.05 Sham vs. PD+Ex), (C) intermediate toe spread (Two-way ANOVA[F = 1.898, p = 0.0651] followed by Bonferroni post hoc test; ***denotes p < 0.001 PD vs. PD+Ex, #denotes p < 0.05, ##denotes p < 0.01, ###denotes p < 0.001 Sham vs. PD) and (D) foot angle (Two-way ANOVA[F = 1.115, p = 0.3683] followed by Bonferroni post hoc test; *denotes p < 0.05, **denotes p < 0.01 PD vs. PD+Ex) in PD in exercise animals . (E) The data from support parameters of each group from post-lesion 2nd to 5th weeks were averaged and plotted indicating that intermediate toe spread improved significantly in PD with exercise animals. (“RPL” One-way ANOVA[F = 7.156, p = 0.0138] followed by Bonferroni post hoc test; #denotes p < 0.05 Sham vs. PD; “RTS” One-way ANOVA[F = 5.134, p = 0.0325] followed by Bonferroni post hoc test; #denotes p < 0.05 Sham vs. PD; “RIT” One-way ANOVA[F = 11.48, p = 0.0033] followed by Bonferroni post hoc test; **denotes p < 0.01 PD vs. PD+Ex, ##denotes p < 0.01 Sham vs. PD; “RFtAng” One-way ANOVA[F = 3.28, p = 0.851] followed by Bonferroni post hoc test) (PL: print length, TS: Toe spread, IT: Intermediate toe spread, Ft Ang: Foot angle)


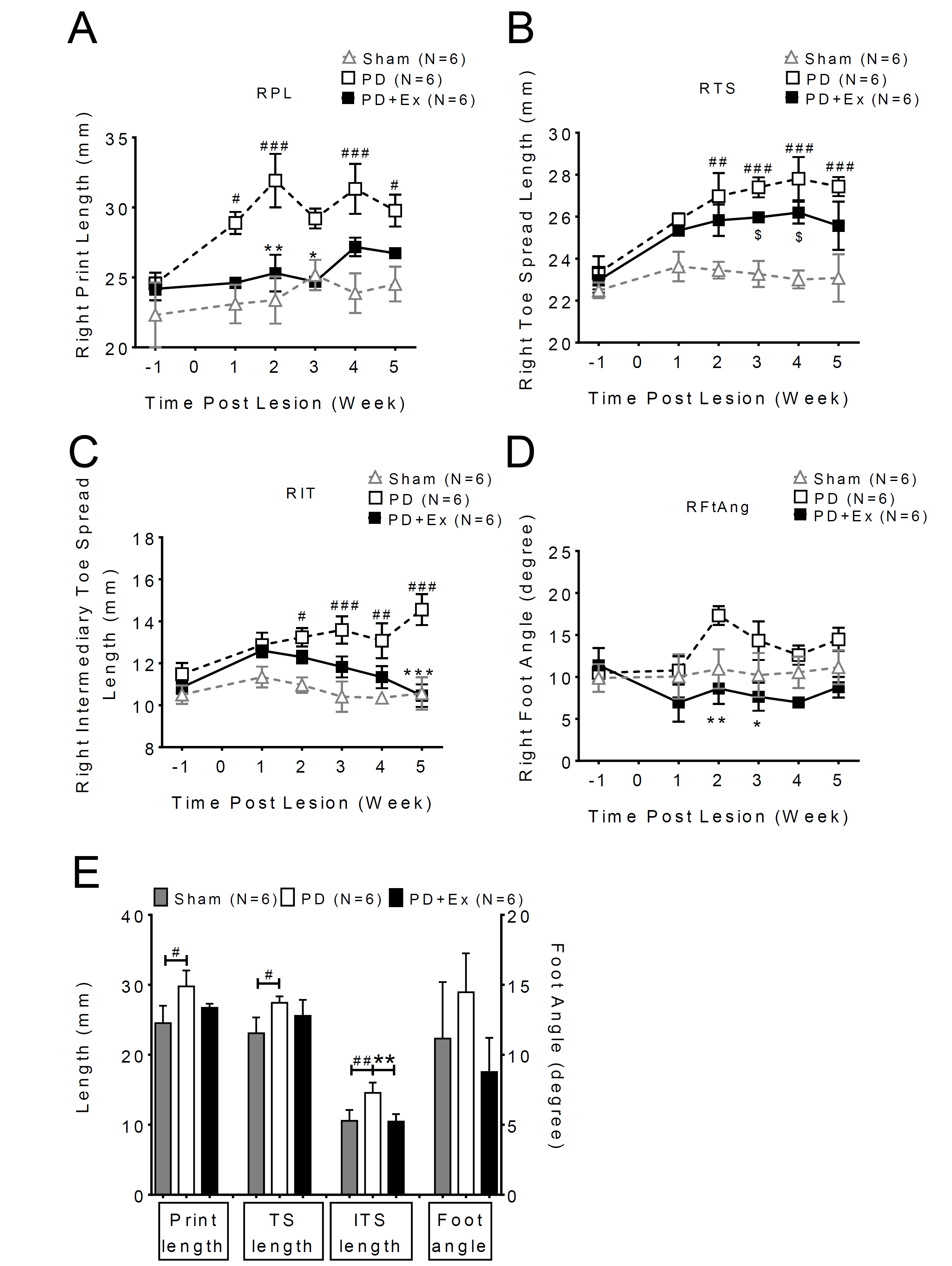

Supplement: Supplementary file 3 — Supplementary data Figure. 2-2 [file 41598_2018_22462_MOESM3_ESM.docx]
