# Supplementary material for: Exercise Ameliorates Motor Deficits and Improves Dopaminergic Functions in the Rat Hemi-Parkinson’s Model
Source: Sci Rep. 2018 Mar 5;8:3973. doi: 10.1038/s41598-018-22462-y (PMC5838260; doi:10.1038/s41598-018-22462-y)

**Exercise Ameliorates Motor Deficits and Improves Dopaminergic Functions in the Rat Hemi-Parkinson's Model**

Yuan-Hao Chen^1*^ MD, PhD, Tung-Tai Kuo^2^ MA, Jen -Hsin Kao^1^ PhD, Eagle Yi-Kung Huang^3^ PhD, Tsung-Hsun Hsieh^4^ PhD, Yu-Ching Chou^5^ PhD, Barry J Hoffer^6, 7^ MD, PhD

^1^Department of Neurological Surgery, Tri-Service General Hospital, National Defense Medical Center, Taipei, Taiwan, R.O.C.

^2^ Graduate Institute of Computer and Communication Engineering, National Taipei University of Technology, Taipei, Taiwan, R.O.C.

^3^ Department of Pharmacology, National Defense Medical Center, Taipei, Taiwan, R.O.C

^4^ Department of Physical Therapy and Graduate Institute of Rehabilitation Science, Chang Gung University, Taoyuan, Taiwan

^5^ School of Public Health, National Defense Medical Center, Taipei, Taiwan. , R.O.C

^6^ Graduate Program on Neuroregeneration, Taipei Medical University, Taipei, Taiwan.

^7^Department of Neurosurgery, Case Western Reserve University School of Medicine, Cleveland, Ohio, USA

*Corresponding author: Yuan-Hao Chen

E-mail: [chenyh178@gmail.com](mailto:chenyh178@gmail.com) (Y-HC)

**Supplementary data Figure.2-3**

The temporal parameters of gait in PD animals also improved on the healthy side (Right side) after exercise. The temporal indices include: (A) stance time (STP) (Two-way ANOVA[F = 1.821, p = 0.0793] followed by Bonferroni post hoc test; ***denotes p < 0.001 PD vs. PD+Ex, #denotes p < 0.05, ##denotes p < 0.01 Sham vs. PD), (B) wing phase (SWP) (Two-way ANOVA[F = 1.175, p = 0.3273] followed by Bonferroni post hoc test; *denotes p < 0.05 PD vs. PD+Ex, ##denotes p < 0.01 Sham vs. PD) and (C) double support (DS) (Two-way ANOVA[F = 1.22, p = 0.299] followed by Bonferroni post hoc test; *denotes p < 0.05, **denotes p < 0.01 PD vs. PD+Ex, #denotes p < 0.05 Sham vs. PD) were improved in PD with exercise animals compared with PD only animals. (D) The data from each group, collected from the 2nd to 5th week, were averaged, showing significant improvement of temporal indices in stance phase on the right side of exercise animals. (“RSTP” One-way ANOVA[F = 6.996, p = 0.0147] followed by Bonferroni post hoc test; *denotes p < 0.05 PD vs. PD+Ex, #denotes p < 0.05 Sham vs. PD; “RSWP” One-way ANOVA[F = 4.016, p = 0.0567] followed by Bonferroni post hoc test; RDS” One-way ANOVA[F = 0.3166, p = 0.7364] followed by Bonferroni post hoc test )


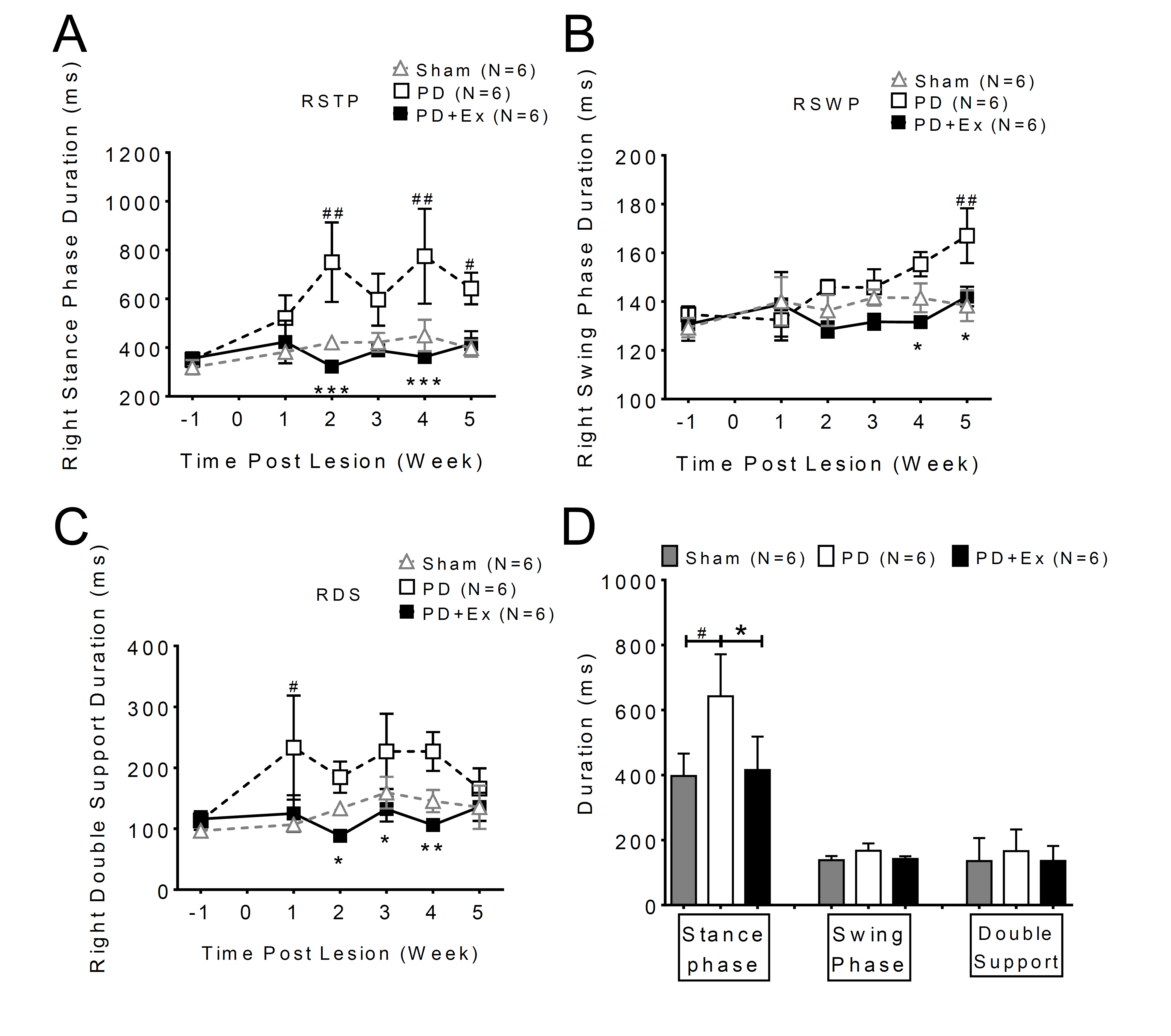

Supplement: Supplementary file 4 — Supplementary data Figure. 2-3 [file 41598_2018_22462_MOESM4_ESM.docx]
