# Supplementary material for: Exercise Ameliorates Motor Deficits and Improves Dopaminergic Functions in the Rat Hemi-Parkinson’s Model
Source: Sci Rep. 2018 Mar 5;8:3973. doi: 10.1038/s41598-018-22462-y (PMC5838260; doi:10.1038/s41598-018-22462-y)
Supplement: Supplementary file 5 — Supplementary data Figure. 3 [file 41598_2018_22462_MOESM5_ESM.pdf]

Exercise Ameliorates Motor Deficits and Improves Dopaminergic Functions in the Rat Hemi-Parkinson's Model

Yuan-Hao Chen[1*] MD, PhD, Tung-Tai Kuo[2] MA, Jen -Hsin Kao[1] PhD, Eagle Yi-Kung Huang[3] PhD, Tsung-Hsun Hsieh[4] PhD, Yu-Ching Chou[5] PhD, Barry J Hoffer[6, 7] MD, PhD

[1]Department of Neurological Surgery, Tri-Service General Hospital, National Defense Medical Center, Taipei, Taiwan, R.O.C.

[2] Graduate Institute of Computer and Communication Engineering, National Taipei University of Technology, Taipei, Taiwan, R.O.C.

[3] Department of Pharmacology, National Defense Medical Center, Taipei, Taiwan, R.O.C

[4] Department of Physical Therapy and Graduate Institute of Rehabilitation Science, Chang Gung University, Taoyuan, Taiwan

[5] School of Public Health, National Defense Medical Center, Taipei, Taiwan. , R.O.C

[6] Graduate Program on Neuroregeneration, Taipei Medical University, Taipei, Taiwan.

[7]Department of Neurosurgery, Case Western Reserve University School of Medicine, Cleveland, Ohio, USA

*Corresponding author: Yuan-Hao Chen

E-mail: chenyh178@gmail.com (Y-HC)

Supplementary data Figure 3 The neuroinfalmmation after 6-OHDA had been ameliorated by 4 weeks force trade mill exercise: To determine the neuroinflammatory reaction after 6-OHDA we performed CD11b stain for microglia activation and GFAP for gliosis reaction survey. (A) The contralateral side striatum data shew no signincant difference between control, 6-OHDA lesion and 6-OHDA-lesion with exercise animal; but the data in lesioned side of striatum (B) indicated that microglia activation induced by 6 –OHDA could be ameliorated by exercise. The same situation could be found by using GFAP staining to evaluate the gliosis. (C) No significant differences between groups in contralateral side striatum. But (D) in lesionded side striatum, the severe gliosis induced by 6-OHDA lesioning while this gliosis could be ameliorated after exercise. (E) To determine the activation of microglia, we quantitated the subtleties of the morphology of the microglia and fractal dimension (Df) of three slides (40×) of each group were measured by Image J with the Fraclac analysis method. The ramified microglia were found to have higher Df values than the activated microglia. The activation of microglia was suppressed by exercise. (One-way ANOVA [$F_{5, 18}$ = 3.367, p=0.0254] followed by Tukey post hoc test; *denotes p < 0.05.). (F) The number of astrocytes were counted and summarized in this panel, which shows gliosis was ameliorated by exercise (ipsilateral side; PD+ ex: Gray bar vs. PD only: black bar, One-way ANOVA [$F_{5, 18}$ = 11.99, p<0.001] followed by Tukey post hoc test; *denotes p < 0.05, **denotes p < 0.01, ***denotes p < 0.01).

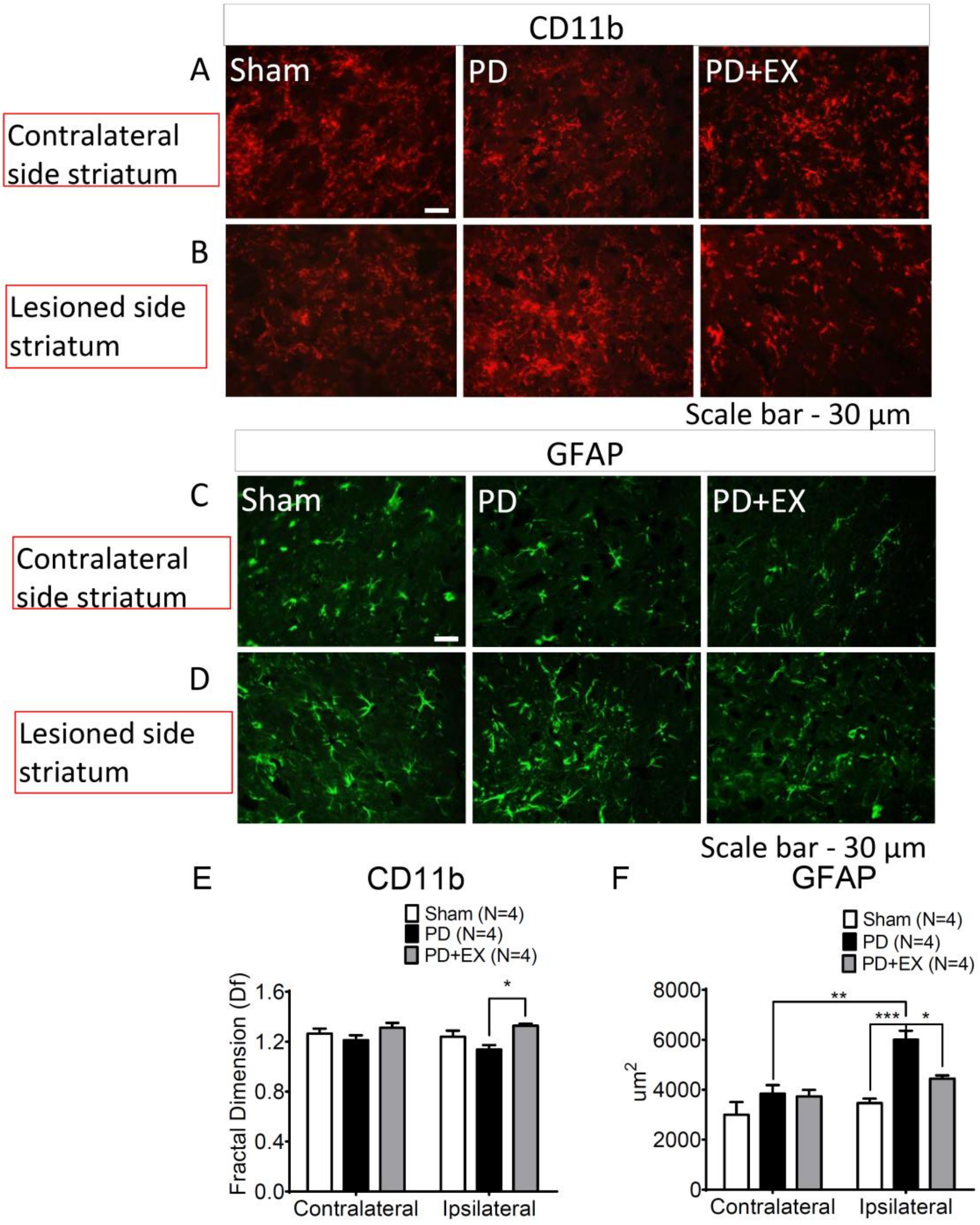
CD11b
A
Sham
PD
PD+EX
Contralateral side striatum
B
Lesioned side striatum
Scale bar - 30 μm
GFAP
C
Sham
PD
PD+EX
Contralateral side striatum
D
Lesioned side striatum
Scale bar - 30 μm
E
CD11b
Sham (N=4)
PD (N=4)
PD+EX (N=4)
Fractal Dimension (Df)
1.6
1.2
0.8
0.4
0.0
*
Contralateral
Ipsilateral
F
GFAP
Sham (N=4)
PD (N=4)
PD+EX (N=4)
um2
8000
6000
4000
2000
0
**
***
*
Contralateral
Ipsilateral
